# Age and sex-dependent sensitivity analysis of a common carotid artery model

**DOI:** 10.1007/s10237-023-01808-0

**Published:** 2024-02-19

**Authors:** Friederike Schäfer, Jacob Sturdy, Leif Rune Hellevik

**Affiliations:** Division of Biomechanics, Department of Structural Engineering, Norwegian University of Science and Technology (NTNU), Richard Birkelands vei 1A, 7034 Trondheim, Norway

**Keywords:** Common carotid artery, Population variations, Uncertainty quantification, Sensitivity analysis

## Abstract

The common carotid artery (CCA) is an accessible and informative site for assessing cardiovascular function which makes it a prime candidate for clinically relevant computational modelling. The interpretation of supplemental information possible through modelling is encumbered by measurement uncertainty and population variability in model parameters. The distribution of model parameters likely depends on the specific sub-population of interest and delineation based on sex, age or health status may correspond to distinct ranges of typical parameter values. To assess this impact in a 1D-CCA-model, we delineated specific sub-populations based on age, sex and health status and carried out uncertainty quantification and sensitivity analysis for each sub-population. We performed a structured literature review to characterize sub-population-specific variabilities for eight model parameters without consideration of health status; variations for a healthy sub-populations were based on previously established references values. The variabilities of diameter and distensibility found in the literature review differed from those previously established in a healthy population. Model diameter change and pulse pressure were most sensitive to variations in distensibility, while pressure was most sensitive to resistance in the Windkessel model for all groups. Uncertainties were lower when variabilities were based on a healthy sub-population; however, the qualitative distribution of sensitivity indices was largely similar between the healthy and general population. Average sensitivity of the pressure waveform showed a moderate dependence on age with decreasing sensitivity to distal resistance and increasing sensitivity to distensibility and diameter. The female population was less sensitive to variations in diameter but more sensitive to distensibility coefficient than the male population. Overall, as hypothesized input variabilities differed between sub-populations and resulted in distinct uncertainties and sensitivities of the 1D-CCA-model outputs, particularly over age for the pressure waveform and between males and females for pulse pressure.

**Supplementary Information:**

The online version contains supplementary material available at 10.1007/s10237-023-01808-0.

## Introduction

Local wall stiffness of the common carotid artery (CCA) is widely recognized as a valuable biomarker useful for prediction of future cardiovascular events and all-cause mortality, and changes in wall stiffness are often a result of pathological disease progression (Vlachopoulos et al. 2010). However, the quantification of arterial wall stiffness is not yet integrated in diagnostic routines (Alastruey et al. 2011; Segers et al. 2020; Nabeel et al. 2020). This is in part due to difficulties in precise measurement of local stiffness, and additionally somewhat imprecise interpretation of measured values of arterial stiffness remains challenging in part due to variability both between individuals and throughout the vascular system. Computational modelling may help overcome both of these challenges, first by linking a model to clinical measurements may enable novel methods for quantifying and interpreting carotid artery stiffness. Additionally, the models may be employed to give context to the interpretation of particular values by exploring the range of variation expected for a given case. Consequently, interpretation of and modelling based on a given value of arterial stiffness must consider the range of stiffness that is likely for a given context. In particular, the age and sex of an individual may imply distinct ranges for arterial stiffness and other parameters influential to the relationship of pressure, flow, and deformation of the arteries. A model which is robust and reliable when applied to one subgroup may require more careful interpretation when applied to other groups as the uncertainties associated with model parameters or typical values are different. These uncertainties result in variability of quantities of interest (QoIs) predicted by the model. This variability must be considered when interpreting these predictions, both for prediction of specific values for an individual case as well as the likely range of values in the relevant population. In this article, we present an example of establishing sub-population specific variations and assessing their impact on a particular model of interest; however, the approach and example of how model output variation depends on sub-population is relevant generally.

Cardiovascular diseases (CVDs) are a leading cause of death globally (Briet et al. 2006) and are generally associated with arterial stiffness (Vlachopoulos et al. 2010; Briet et al. 2006; Blacher et al. 1998; Ferreira et al. 2002). In healthy humans, arterial stiffness increases with increasing age due to structural changes in the arterial wall (Laurent et al. 2006). The ratio between elastin and collagen fibres as well as the three-dimensional architecture, the connectivity between matrix constituents, calcification, and advanced glycation end-product accumulation determine the arterial wall’s structural characteristics (Chirinos 2012). Elastin fibres degrade while the number of collagen fibres and fatty deposits in the walls of large and medium-size arteries increase with ageing, which in turn leads to increased arterial stiffness [11]. Additionally, sex hormones have an impact on cardiovascular pathologies and risk factors associated with arterial stiffness (DuPont et al. 2019). Vascular diseases, like the deposition of plaque in the arterial wall (van de Vosse and Stergiopulos 2011), as well as lifestyle and genetics (Chirinos 2012) can all affect arterial stiffness. The state of an individual’s cardiovascular system may be characterized by the pressure and flow waveform, which depend on the arterial wall stiffness (Alastruey et al. 2011).

A number of arterial stiffness indices have been proposed, for example, the arterial compliance *C*, distensibility coefficient $\textrm{DC}$, stiffness index $\beta$, Young’s modulus *E*, Peterson modulus $E_\textrm{P}$, and pulse wave velocity (PWV) (Boutouyrie et al. 2014). The most commonly used index in diagnostics is the PWV, which is the speed at which a perturbation of pressure propagates through a vessel (Aguado-Sierra et al. 2006). Several methods exist for clinical measurement of PWV, where the gold standard is the carotid-femoral PWV estimating central aortic stiffness (Laurent et al. 2006). However, this measurement estimates only an average arterial wall stiffness of the aorta. CVDs can lead to strong spatial variations of material properties in the arterial walls. Therefore, local properties of an artery’s wall are of interest since they give a closer insight into the current status of an individual’s cardiovascular system. New ultrasound technologies have been developed to determine local arterial stiffness. This equipment is more expensive and validation of this technology is still pending (Segers et al. 2020).

As integration of diverse clinical measurements and estimation of local arterial properties is quite challenging, computational modelling may be a valuable support by providing a novel means to estimate local properties through inverse modelling as well as supporting and improving the interpretation of measurements of both stiffness and haemodynamics. For example, the properties of the CCA and its distal vasculature determine the local dynamics and relationship between pressure, flow and distention for a given inflow which may be measured using Doppler ultrasound. Local modelling may be useful for characterizing and systematizing the relationship between CCA properties and clinically feasible measurements. This in turn may provide a basis for better understanding the relationship between the state of the CCA and overall cardiovascular risk (Chiesa et al. 2019). The CCA’s position as a conduit to the cerebral circulation, its propensity for atherosclerosis, and its ease of access for measurement reinforce the clinical relevance of this artery. In particular, the carotid arteries are known to exhibit a pattern of pathological changes distinct from those found in other arteries in numerous diseases and pharmaceutical interventions (Paini et al. 2007; Bruno et al. 2017; Laurent 1995; Asmar 2007), thus focused local modelling may be an avenue to better and earlier characterize these pathologies as well as gain information of the mechanobiology of their progression.

Computational models of haemodynamics offer a means to link the arterial stiffness at specific regions to haemodynamic indices which may be more directly interpreted; however, these models depend on numerous parameters that must be assumed as they cannot be measured in clinical contexts. We investigate the uncertainties of various model parameters across the population and subsequently evaluate the impact of these uncertainties on the model’s predictions of pressure and deformation. Many model parameters depend on age and sex (DuPont et al. 2019; Charlton et al. 2019; Engelen et al. 1996) such that specific sub-populations may have distinct model parameter distributions leading to different model performance for different population groups. This work aims to better characterize the influence of variability in arterial stiffness and other assumed model parameters on deformation of the CCA, as this deformation is an ideal target for inverse modelling-based estimation of arterial wall properties. Accounting for model parameter uncertainties due to measurement errors, lack of knowledge, and variations in the population is a challenge facing most biomedical modelling efforts. As such, the process we applied to characterize these uncertainties is also relevant beyond the specific application we present, as a thorough characterization of uncertainties based on existing evidence of population variability, particularly in specific sub-populations, greatly enhances the value of such analyses.

To the authors’ knowledge, there exists one review for reference values for age and sex groups for carotid artery distension, diameter, and $\textrm{DC}$ (Engelen et al. 1996) and a summary of literature findings for diverse cardiovascular parameters as a function of age (Charlton et al. 2019). However, both works are based on sub-populations without any risk factors. This is very limiting since the presence of risk factors and cardiovascular morbidity increases with increasing age. So far, no age and sex-dependent reference intervals for a general cross-sectional population of geometric and material parameters of the CCA exist.

The aim of this work was to determine intervals of geometric and material properties for a cross-sectional population dependent on sex and age groups based on a structured literature review. These distributions were then propagated through a numerical 1D-model of the CCA to investigate the influence of age and sex on the distribution of sensitivity indices for each quantity of interest. Such an analysis is a step in assessing the further development of methods to link numerical models to clinical data. Further, characterizing model output variability is essential for bringing numerical modelling into clinical practice as well as in the certification of medical devices, because model credibility needs to be demonstrated through verification, validation, and uncertainty quantification (UQ) (Anderson et al. 2007).

## Methods

**Fig. 1:**
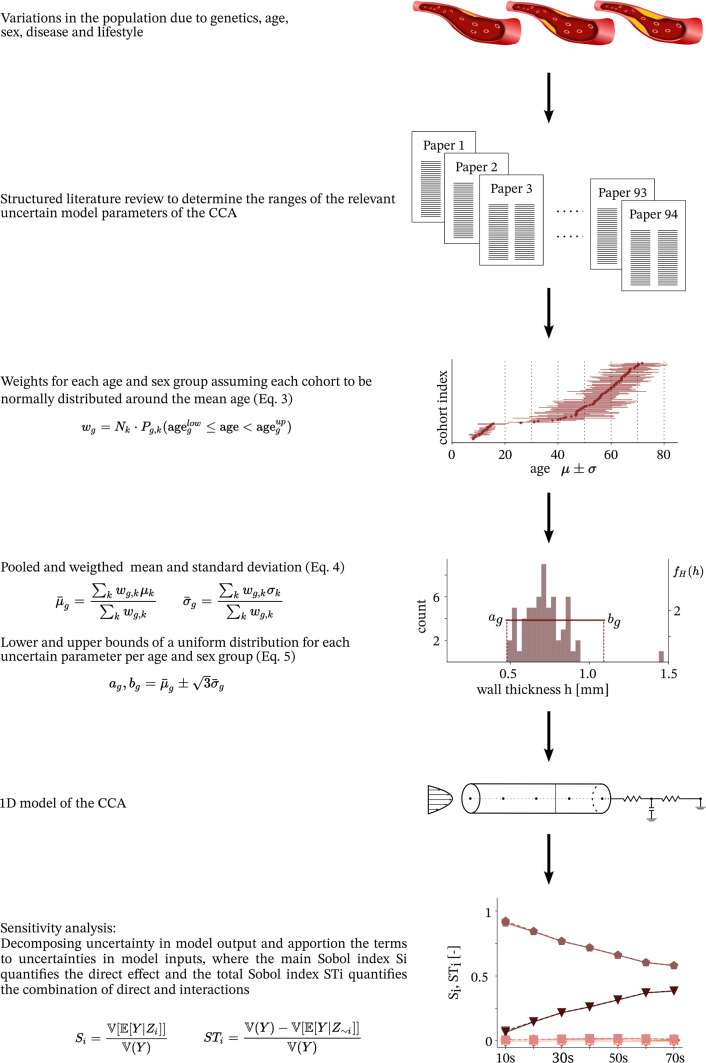
Overview of the workflow

We investigated the age and sex-dependent sensitivity structure of a 1D-model of the CCA. We use the term sensitivity structure of a numerical model is the distribution of sensitivity indices for a specific QoI. Each subject population has its own model input variations which may lead to a different distribution of model output sensitivity, that is a change in the sensitivity structure between populations. A structured literature review established age and sex specific variabilities for model input parameters. Using polynomial chaos (PC) expansion, UQ and sensitivity analysis (SA) were performed. Figure 1 visualizes the workflow of this study.

### Literature review

To investigate the age and sex-specific sensitivity of a 1D-model of the CCA with respect to uncertainties in the input parameters, ranges for each age and sex group were determined through a structured literature review. The literature review’s scope was defined following the PICO framework where the details are shown in Table 1. The structured literature review was performed following Cochrane’s Handbook for Systematic Reviews and Interventions guidelines (Forero et al. 2019; Higgins et al. 2022).

**Table 1:** PICO framework to define the scope of the literature search

Concept	Definition
Population	Representing a general population, meaning that all ages (7–90 years) are considered, all fitness levels, and body sizes; subjects may show risk factors like hypertension, diabetes, smoking, atherosclerosis, overweight, stenosis and aneurysms; studies considering a population suffering from a rare disease which changes significantly cardiovascular mechanics were excluded
Intervention	Observational and interventional studies; however, in interventional studies only measurements from the control group and the intervention group before the intervention were considered
Comparison	Quantify the difference of relevant haemodynamic parameters of the CCA between sex and age groups
Outcome	Identify differences in haemodynamic parameters with respect to sex and age; evaluate mean and standard deviations for each age and sex group which can be used for UQ and SA of th 1D-numerical model of the CCA

***Eligibility criteria***

Published studies had to fulfil a set of eligibility criteria in order to be included in the literature review. These eligibility criteria were:

- The publication must be a peer-reviewed journal article or conference paper.
- The results must be original, thus, literature reviews based on earlier published data were excluded. However, the review’s references were used for identifying further relevant publications. If several publications were based on the same data set, only one study was considered.
- Language of the full-text publication was restricted to English.
- The publication’s full-text had to be either openly accessible or available through the library services from the Norwegian University of Science and Technology and be published before the 1st of December 2022.
- The scope of the studies had to be on arterial stiffness of the CCA in humans.
- Measurements had to be performed through noninvasive means.
- Observational and interventional studies were considered. In the case of interventional studies, only data from the control group and pre-intervention data were eligible for the review.
- All study time frames were considered; several months to longitudinal studies lasting for more than a decade.
- Studies investigating the influence of rare diseases and with severe implications on cardiovascular parameters were excluded.

**Fig. 2:**
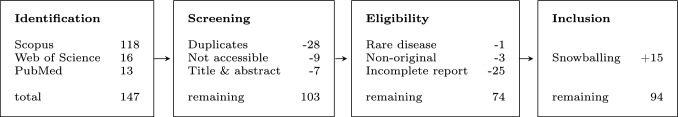
PRISMA diagram of the publication selection process showing the number of publications considered in each stage of the review

From the study requirements, a search string filtering for relevant publications was constructed. The three online databases Scopus, Web of Science and PubMed were searched for publications with the following search string: TITLE-ABS-KEY (“common carotid artery” AND “stiffness” AND “Young’s modulus” AND “measure*”). Scopus and Web of Science cover a wide spectrum of literature, whereas PubMed focuses on medical content. We selected “Young’s modulus” as a keyword in the search string since the numerical 1D-model uses this parameter to describe the material properties of the arterial wall. Following the Preferred Reporting Items for Systematic Reviews and Meta-Analyses (PRISMA), a flow diagram is depicted in Fig. 2, reporting the number of publications in the identification, screening, eligibility, and inclusion steps of the literature review process. In the screening process duplicates, non-accessible, and publications not following the eligibility criteria from their title and abstract were removed. The full-text of all remaining publications’ was considered for data extraction. Publications with non-original data sets, or incomplete data, measurement, or data analysis protocols were excluded. Cohorts containing only subjects with Ehlers-Danlos and Williams Syndrome were excluded due to the syndrome’s limited occurrence and its significant influence on cardiovascular changes. We searched the bibliography of excluded review papers and analysed full texts for further relevant studies not identified by the search string. Thus, 15 additional publications were included. Table 2 shows all the labels for which data was extracted from the publications. If a study included measurements from the left and right CCA for each subject, then only the data from one side was extracted. Relevant data for uncertainty propagation were age, sex, blood pressure, geometric parameters of the CCA, and arterial stiffness measures of the study population. If possible, non-reported values were computed from the reported data. Python was used to perform data analysis.

***Data pooling***

Literature data was grouped by sex and age. With respect to sex, the data was categorized as male, female, or mixed in cases where no separate data for the two sexes was reported. The data was split into age groups by decade with the youngest group ranging from ages seven to 20. The majority of cohorts included individuals from multiple age groups but reported only summary statistics. To account for this spread, data was pooled using weights which took this into account. Per cohort a weight $w_g$ was computed for each age group which was based on cohort size $N_k$, $k \in [1, 2,..., K]$ cohorts, and the probability $P_{g,k}$ of an individual of this cohort belonging to this specific age group *g*, $g \in [<\text {20, 20 s,..., } <\text {70}]$ such that

1 $$\begin{aligned} w_g = N_k \cdot P_{g,k} \left( \text {age}_g^{\text {low}} \le \text {age} < \text {age}_g^{\text {up}}\right) . \end{aligned}$$

The probability was calculated by assuming a normal distribution with respect to age within each cohort using the reported mean and standard deviation. For each age and sex group, a pooled mean $\overline{\mu }_g$ and standard deviation $\overline{\sigma }_g$ was computed using the weights from Eq. (1)

2 $$\begin{aligned} \overline{\mu }_g = \frac{\sum _{k = 1}^{K} w_{g,k} \mu _k}{\sum _{k = 1}^{K} w_{g,k} }, \quad \overline{\sigma }_g = \frac{\sum _{k = 1}^{K} w_{g,k}~\sigma _k}{ \sum _{k = 1}^{K} w_{g,k} } \end{aligned}$$

with $\mu _k$ and $\sigma _k$ as respective cohort mean and standard deviation.

Due to the lack of knowledge on the underlying probability distribution of each parameter, lower (*a*) and upper (*b*) bounds of a uniform distribution were computed based on the pooled mean and standard deviation,

3 $$\begin{aligned} \overline{\sigma }_g = \sqrt{\frac{(b_g-a_g)^2}{12}} \hspace{1cm} \text{ such } \text{ that } \hspace{0.5cm} a_g,b_g = \overline{\mu }_g \pm \sqrt{3}\, \overline{\sigma }_g. \end{aligned}$$

In solid mechanics, the Young’s modulus is one of the most common stiffness measures. However, this is the least common stiffness index measured in a clinical setting. Thus, the literature review did not provide Young’s modulus values for each age and sex group. As there was significantly more data available for $\textrm{DC}$, which also is a stiffness measure which can be related to the Young’s modulus though the $\textrm{PWV}$, we used $\textrm{DC}$ as an uncertain input parameter during UQ and SA and computed the Young’s modulus from $\textrm{DC}$, *h* and *D* which were sampled from the distributions determined from pooled values. Following the Moens–Korteweg equation (Chirinos 2012), the $\textrm{PWV}$ is related to the Young’s modulus *E* as

4 $$\begin{aligned} \textrm{PWV} = \sqrt{\frac{E h}{D \rho }}, \end{aligned}$$

where *h* is the vessel wall thickness, *D* the vessel diameter, and $\rho$ the blood density. Chirinos (2012) established a relation for the $\textrm{PWV}$ in terms of $\textrm{DC}$ as

5 $$\begin{aligned} \textrm{PWV} = \sqrt{\frac{1}{\rho }\frac{1}{\textrm{DC}}}. \end{aligned}$$

Combining Eqs. (4) and (5) yields $E~=~\frac{D}{h}~\frac{1}{\textrm{DC}}$.

We did not discriminate between lumen diameter and mean diameter reported in the studies. We also have not adjusted mean values to represent lumen diameter because of lack of knowledge on how to base such a correction parameter for a diverse set of protocols and measurement equipment. Further, in the uncertainty propagation, we assume that the input parameters are independent from one another, and thus, there is no dependency between vessel diameter and wall thickness. This means that the variations of the lumen diameter are assumed proportional to the variations in the mean diameter leading to the same proportionality of uncertainty.

Since age and sex-dependent reference values for *D*, $\Delta D$, and $\textrm{DC}$ were previously reported for a healthy population, the pooled parameters from the literature review were compared with those reference values (Engelen et al. 1996). To investigate effects due to variations within a healthy population against general population variations, we performed UQ and SA twice for each group. First, uncertainties in *D* and $\textrm{DC}$ were based on the reference values of the healthy population (Engelen et al. 1996). Subsequently, uncertainties in *D* and $\textrm{DC}$ were based on values found during the literature review.

**Table 2:** Description of the extracted data from the literature

Data label	Unit	Description
Location of study	–	Country where the study participants were recruited; if not specified then the country of the first author’s institution was assumed as the study location
Sample size *N*	–	Number of subjects in the cohort
Age	years	Mean and standard deviation of the participant’s age
Sex	%	Percentage of females in the study
BMI	–	Mean and standard deviation of the body mass index (BMI)
$P_{\textrm{sys}}$	mmHg	Mean and standard deviation of the systolic brachial blood pressure measured in a non-invasive way (e.g. cuff)
$P_{\textrm{dia}}$	mmHg	Mean and standard deviation of the diastolic brachial blood pressure measured in a non-invasive way (e.g. cuff)
$\textrm{PP}$	mmHg	Mean and standard deviation of the pulse pressure; computed as $\text {PP} = P_{\textrm{sys}} - P_{\textrm{dia}}$
IMT	mm	Mean and standard deviation of the intima-media thickness measured through non-invasive means (e.g. ultrasound)
$D_{\textrm{sys}}$	mm	Mean and standard deviation of the systolic lumen diameter of the CCA
$D_{\textrm{dia}}$	mm	Mean and standard deviation of the diastolic lumen diameter of the CCA
*D*	mm	Mean and standard deviation of the mean lumen diameter of the CCA; evaluated as $D = {1}/{2} (D_{\textrm{sys}} + D_{\textrm{dia}})$
$\Delta D$	mm	Mean and standard deviation of the distension of the CCA over one heart cycle; evaluated as $\Delta D~=~D_{\textrm{sys}}~-~D_{\textrm{dia}}$
$\epsilon$	–	Mean and standard deviation of the strain in the vessel; evaluated as $\epsilon = \frac{D_{\textrm{sys}} - D_{\textrm{dia}}}{D_{\textrm{dia}}}$
*E*	kPa	Mean and standard deviation of the Young’s/incremental elastic modulus; evaluated as $E = 3 \cdot \frac{1 + \frac{D_{\textrm{dia}}}{4} \pi }{\pi \left( \frac{D_{\textrm{dia}}}{2}+\textrm{IMT}\right) ^2 - \pi \left( \frac{D_{\textrm{dia}}}{2}^2\right) } \cdot \frac{D_{\textrm{sys}}^2 - D_{\textrm{dia}}^2}{D_{\textrm{dia}}^2~\textrm{PP}}$
$\beta$	–	Mean and standard deviation of the $\beta$ stiffness index; evaluated as $\beta = \frac{ln\left( \frac{P_{\textrm{sys}}}{P_{\textrm{dia}}}\right) }{\frac{D_{\textrm{sys}}- D_{\textrm{dia}}}{D_{\textrm{dia}}}}$
$E_{\textrm{P}}$	kPa	Mean and standard deviation of the Peterson index; evaluated as $E_P = \frac{\textrm{PP}}{\frac{D_{\textrm{sys}}- D_{\textrm{dia}}}{D_{\textrm{dia}}}}$
$\textrm{DC}$	$10^{-3}$ kPa^-1^	Mean and standard deviation of the distensibility coefficient; evaluated as $\textrm{DC} = \frac{D_{\textrm{sys}}^2 - D_{\textrm{dia}}^2}{D_{\textrm{dia}}^2 \textrm{PP}}$
$\textrm{PWV}$	m/s	Mean and standard deviation of the carotid-femoral pulse wave velocity (PWV)
Risk factors	%	Percentage of a certain risk factor present in the study population; the considered risk factors were diabetes, atherosclerosis, aneurysms, chronic kidney disease and dialysis patient, hypertension, obesity, past or current smoker, hyperlipidaemia

### 1D-model of the CCA

**Fig. 3:**
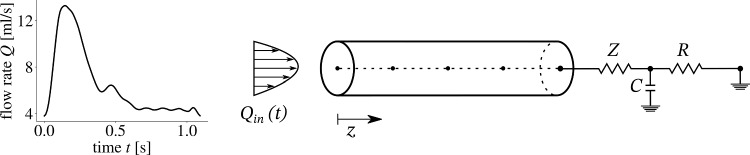
Representation of the 1D-model of the CCA. A parabolic inflow was prescribed at the inlet with a representative flow rate and waveform (Figueroa et al. 2006) and at the outlet a three-element Windkessel model mimicked the behaviour of the downstream vasculature with the electrical elements analogues of arterial impedance *Z*, compliance *C*, and resistance *R*

As shown in Fig. 3, the CCA was modelled as a straight, deformable tube with *z* as the axial coordinate along the vessel. The cross-sectional averaged pressure *P*, flow rate *Q*, and diameter change $\Delta D$ were evaluated at five equidistant points along the centreline. It was shown through a convergence study that pressure *P*, flow rate *Q*, and diameter change $\Delta D$, did not change for an increase in spatial points. Blood flow was modelled as an axisymmetric and laminar flow of an incompressible Newtonian fluid with dynamic viscosity $\mu$. The vessel wall deformed purely in the circumferential direction. Further, the wall was modelled as an impermeable and homogeneous material. Following these assumptions, the conservation of mass and momentum were

6a $$\begin{aligned} \frac{\partial A}{\partial t} + \frac{\partial (Au)}{\partial z}&= 0 \end{aligned}$$

6b $$\begin{aligned} \frac{\partial u}{\partial t} + u \frac{\partial u}{\partial z} + \frac{1}{\rho } \frac{\partial P}{\partial z}&= \frac{f}{\rho A}, \end{aligned}$$

with the time *t*, cross-sectional area *A*, cross-sectional average velocity *u*, fluid density $\rho$, and the frictional force term per unit length *f*. This force term accounted for the wall shear stress and convective inertia terms and it’s magnitude depended on the fluid flow’s velocity profile described with a symmetric polynomial velocity model as

7 $$\begin{aligned} u_{r}(z, r, t) = u(z,t) \frac{\zeta + 2}{\zeta } \left[ 1- \left( \frac{r}{R}\right) ^{\zeta } \right] . \end{aligned}$$

The velocity $u_r$ at a given radial distance *r* from the centreline depended on the vessel radius R and the shape of the velocity profile described by the polynomial order $\zeta$, where $\zeta = 2$ gave a parabolic profile and the friction term becomes $f~=~-2(\zeta ~+~2)~\mu ~\pi$.

The arterial wall was modelled as a thin, incompressible, homogeneous, isotropic, and elastic material. Interaction between the blood flow and the vessel wall was described by the tube law (Sherwin et al. 2003) relating the pressure inside the vessel to the lumen cross-sectional area as

8 $$\begin{aligned} P = P_{\textrm{dia}} + \frac{\beta }{A_{\textrm{dia}}} \left( \sqrt{A} - \sqrt{A_{\textrm{dia}}}\right) \hspace{0.3cm} \text{ with } \, \, \beta = \frac{\sqrt{\pi } E h}{(1-\nu ^2) }. \end{aligned}$$

$A_{\textrm{dia}}$ and $P_{\textrm{dia}}$ were the diastolic cross-sectional area and pressure, and the material properties were described with the Young’s modulus *E*, the wall thickness *h*, and the Poisson ratio $\nu$.

As an inlet boundary condition, a representative CCA flow rate and waveform (Figueroa et al. 2006) was prescribed with a parabolic profile, thus $\zeta ~=~2$. Flow rate and waveform of blood in the CCA can be measured in a clinical setting and was assumed to be known for this study. At the outlet, the 1D model was coupled with a three-element Windkessel model that imitated the behaviour of the downstream vasculature, largely the cerebral vessels. The first resistor *Z* in the electrical analogue modelled the arteries characteristic impedance, and the following resistor *R* and capacitor *C* represented the resistance and compliance of the vessels distal to the CCA, primarily the cerebral circulation. Flow rate and pressure were related in the Windkessel model as

9 $$\begin{aligned} \frac{\partial P}{\partial t} + \frac{P}{\text{RC}} = \left( \frac{1}{C} + \frac{Z}{\text{RC}} \right) \, Q + Z \, \frac{\partial Q}{\partial t}. \end{aligned}$$

The system of equations (Eqs. (6), (8), and (9)) was solved with an explicit MacCormack scheme, which is second order in space and time (Boileau et al. 2015).

### Sensitivity analysis

Lack of knowledge, measurement errors, as well as biological and pathological variations lead to uncertainties in the input parameters used in numerical models of blood vessels (Anderson et al. 2007). Quantifying the distribution of the model output *Y* due to uncertain inputs is necessary for model validation and for a model’s integration into clinical decision-making (Huberts et al. 2018). SA informs about the contribution of particular uncertain input parameters and their interactions to model output variability (Eck et al. 2016). PC expansion is an efficient method for performing UQ and SA (Eck et al. 2016).

In a deterministic setting, the function *f* relates the deterministic inputs *z* with the deterministic model output *y*

10 $$\begin{aligned} y = f({\textbf {z}}). \end{aligned}$$

When uncertainties in the input parameters are considered then the model becomes stochastic. The function *f* relates then a vector of input variables ${\textbf {Z}}$ to the stochastic output *Y*

11 $$\begin{aligned} Y = f({\textbf {Z}}). \end{aligned}$$

In PC expansion, model output *Y* is approximated through the sum of a finite number *N* of polynomials

12 $$\begin{aligned} Y \approx \sum _{p=0}^{N} c_p \hspace{1mm} \Phi _p(\varvec{Z}), \end{aligned}$$

where $c_p$ are expansion coefficients and $\Phi _p$ are orthogonal polynomials depending only on the independent random inputs ${\textbf {Z}}$. The distribution type of the random inputs $Z_i$ determines the orthogonal polynomials following the Wiener-Askey scheme. Expansion coefficients $c_p$ were evaluated with a regression approach, where the $L^2$-normed difference between a set of model evaluations and the PC expansion was minimized. Stable least square minimization required an overdetermined system. Therefore, twice as many samples as number of coefficients in the truncated polynomial were evaluated for computing $c_p$ (Eck 2016).

Statistical moments and variance-based sensitivity measures can be computed analytically from the PC expansion. Total variance of model output ${\text {Var}}[Y]$ was approximated with the total variance of the PC expansion output ${\text {Var}}[Y_{\text{PC}}]$ as

13 $$\begin{aligned} {\text {Var}}[Y] \approx {\text {Var}}[Y_{\text{PC}}] = \sum _p {\text {Var}}[c_p \hspace{1mm} \Phi _p (\varvec{Z})]. \end{aligned}$$

The main Sobol index $S_i$ is a global, variance-based measure which quantifies a particular input parameter $z_i$’s contribution to total model output variance (Saltelli et al. 2008). It can readily be computed from the PC expansion as the fraction of output variance due to $z_i$ with respect to the total model output variance:

14 $$\begin{aligned} S_i \approx \frac{1}{{\text {Var}}[Y_{\text {PC}}]} \sum _{p \in A_i} {\text {Var}}[c_p \hspace{1mm} \Phi _p], \end{aligned}$$

where the set $A_i$ indexes all basis functions only dependent on $z_i$. To quantify the effect of model parameter interactions, the total Sobol index $\text {ST}_i$ relates the total model output variance to the variance of parameter $z_i$ and all its interactions with $z_ {\sim i}$. With the set of all basis functions depending on $z_i$ indexed by $A_{T,i}$, $\text {ST}_i$ can then be computed:

15 $$\begin{aligned} \text {ST}_i \approx \frac{1}{{\text {Var}}[Y_{\text {PC}}]} \sum _{p \in A_{T,i}} {\text {Var}}[c_p \hspace{1mm} \Phi _p]. \end{aligned}$$

If $S_i \approx \text {ST}_i$ then no significant interaction effects between the uncertain input parameters are present in the model. For quantities of interest that vary over time, a time averaged sensitivity index is useful to characterize the overall influence of parameters (Eck et al. 2017). This may be achieved for the main sensitivity index by

16 $$\begin{aligned} \text {TAS}_i = \frac{ \sum _{k=1}^{n} S_i^k{\text {Var}}{[Y_{\text {PC}}(t_k)]}}{\sum _{k=1}^{n}{\text {Var}}{[Y_{\text {PC}}(t_k)]}}, \end{aligned}$$

and averaged total sensitivity indices are

17 $$\begin{aligned} \text {TAST}_i = \frac{ \sum _{k=1}^{n} \text {ST}_i^k{\text {Var}}{[Y_{\text {PC}}(t_k)]}}{\sum _{k=1}^{n} {\text {Var}}{[Y_{\text {PC}}(t_k)]}}. \end{aligned}$$

In the results, we use the notation $S_i$ and $\text {ST}_i$ for both, but where the quantity of interest is time varying it is implied that the sensitivity was computed by Eq. (16) or (17).

In this work, we considered a total of eight uncertain input parameters, which were the fluid properties of density $\rho$, and viscosity $\mu$, wall properties of the wall thickness *h*, Poisson ratio $\nu$, and distensibility coefficient $\textrm{DC}$, which was used to compute the Young’s modulus according to Eq. (5), lumen diameter *D*, and in the Windkessel model compliance *C* and total arterial resistance $R_{\text {tot}} = Z + R$. Mean values of *Z* and *R* were $2.4875 \cdot 10^8$ Pa s m^-3^ and $1.8697 \cdot 10^9$ Pa s m^-3^, respectively (Xiao et al. 2014). Since there is little evidence that $\rho$, $\mu$, and $R_{\mathrm {\text {tot}}}$ vary between different age groups and sexes, these parameters were considered to be age and sex independent (Charlton et al. 2019; Irace et al. 2012; Kenner 1989). Compliance decreases with increasing age, and thus, *C* was adjusted from a 25-year-old reference value of $1.7529 \cdot 10^{-10}$ m^3^ Pa^-1^ following $1.7529 \cdot 10^{-10} \cdot (128.4-1.136 \cdot \text {age})/100$ (Charlton et al. 2019) to represent the respective age group. No sex discrimination was applied. Uncertainties in the Windkessel model parameters were assumed to be within ±20 % from their respective mean values because of lack of measurements and knowledge. Age and sex-dependent parameters were *D*, $\textrm{DC}$, and *h*. Vessel length was kept constant at 126 mm.

To confirm convergence of sensitivity indices, PC expansion was computed for orders one to three, with a total number of samples of 18, 90, and 330, respectively, such that the largest difference between orders for any sensitivity index was less than 0.016. Main and total Sobol indices were computed for the last cardiac cycle at the mid-point of the artery. The QoIs in the SA were the diameter change $\Delta D$, the pressure *P*, and the pulse pressure $\textrm{PP}$. The indices for $\Delta D$ and *P* were summarized over time by a variance-weighted average over the cycle (Eqs. (16) or (17) (Eck et al. 2017)).

## Results

Main findings from the literature review are shown and comparison of pooled data of *D*, $\Delta D$, and $\textrm{DC}$ with reference values from a healthy sub-population are displayed (Engelen et al. 1996). Further, the results of the age and sex informed UQ and SA for a healthy sub-population, a general population based on the literature review, and UQ and SA without sex discrimination but age dependence are presented.

### Literature review

**Fig. 4:**
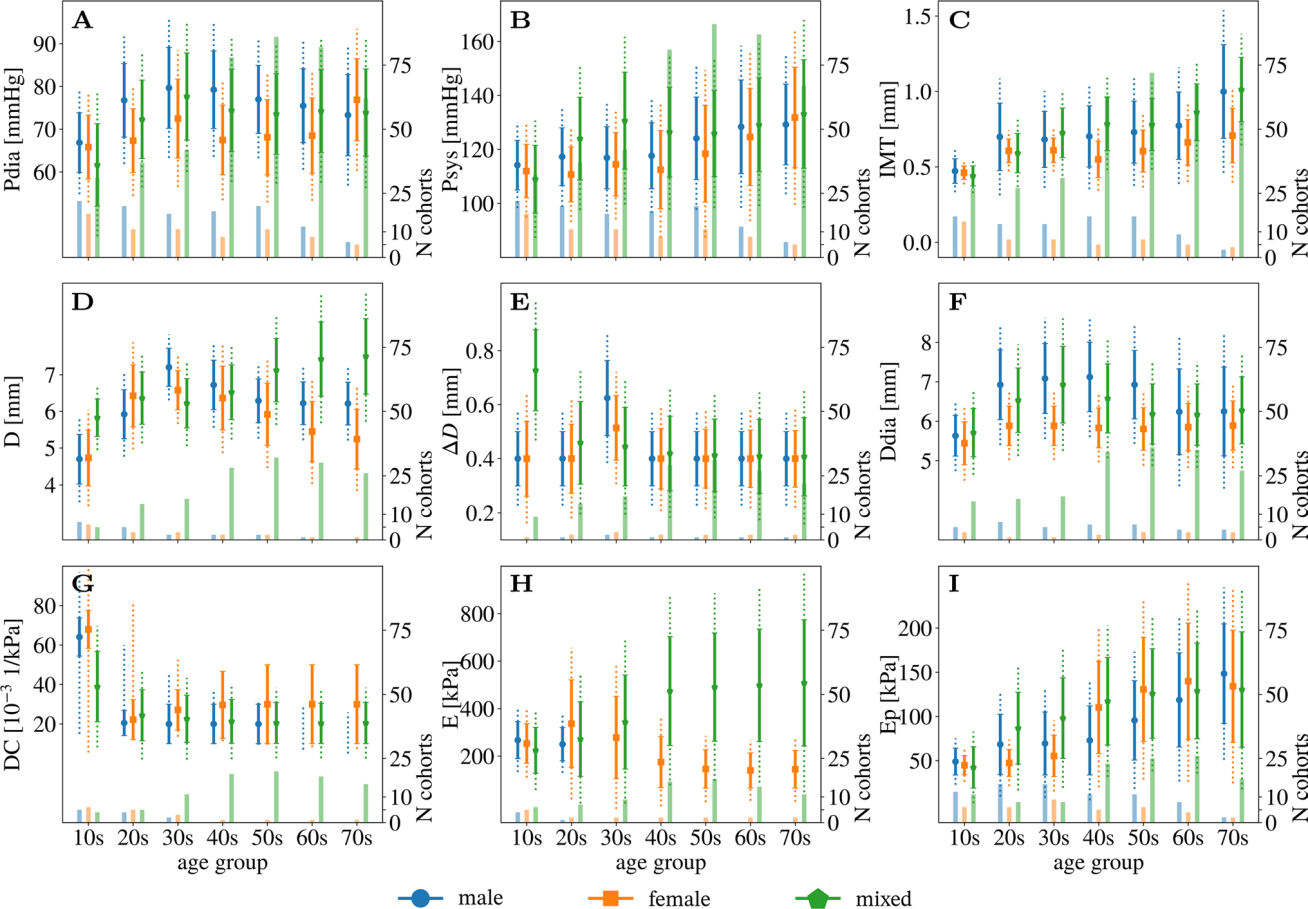
Visualization of pooled mean (Eq. (2); circle, square, and pentagon for male, female, mixed group, respectively), standard deviation (Eq. (2); solid line) and range (Eq. (3); dotted line) for the parameters **A** $P_{\textrm{dia}}$, **B** $P_{\textrm{sys}}$, **C** $\textrm{IMT}$, **D** $D_{\textrm{mean}}$, **E** $\Delta D$, **F** $D_{\textrm{dia}}$, **G** $\textrm{DC}$, **H** *E*, **I** $E_{\textrm{P}}$ for every indicated age and sex group. The bars at the base of each figure indicate the number of cohorts from which data was included in the pooling

Figure 4 displays the pooled mean and one standard deviation of $P_{\textrm{dia}}$, $P_{\textrm{sys}},$ geometric parameters $\textrm{IMT}$, *D*, $\Delta D$, $D_{\textrm{dia}}$, and stiffness indices $\textrm{DC}$, *E*, and $E_{\textrm{P}}$ for each age and sex group. The lower and upper boundaries of a uniform distribution with the same mean and standard deviation of each parameter are marked through dashed lines. The number of available cohorts for each mean age and sex group as well as its subject characteristics is summarized in Table 3. However, as not every study evaluated or reported each parameter of interest, the number of cohorts used to compute pooled mean and standard deviation varied for each parameter, thus, the number of cohorts contributing to each pooled value is indicated by the bars below the respective mean and standard deviation. Overall,

**Table 3:** Number of cohorts available for each mean age and sex group and total number of subjects available for each age and sex group. However, not every study evaluated or reported each parameter of interest. The last column summarizes the subject characteristics of each group with the number of cohorts given in parentheses. Abbreviations are cross-sectional study (CSS), healthy subjects (HS), chronic kidney disease (CKD), hypertension (HT), diabetes (DI), cardiac disorder (CD), and smoking (SMK).

Age	Sex	Cohorts	# Subjects	% Females	Subject characteristics
10 s	Male	14	757	0	CSS (8), HS (2), overweight/obese children (4)
	Female	14	826	100	CSS (8), HS (2), overweight/obese children (4)
	Mixed	21	1213	39.7	CSS (1), CS (10), overweight/obese (10), DI (1), HT (1), CKD (1), arthritis (1), poor growth as foetus (1), congenital heart defect (2), dyslipidemia (1)
20 s	Male	5	116	0	CS (1), HS (1), high cardiorespiratory fitness (2), recreationally active men (1)
	Female	2	179	100	CSS (1), HS (1)
	Mixed	4	73	37.5	HS (4)
30 s	Male	10	4282	0	CS (4), DI (3), SMK (3)
	Female	7	906	100	CSS (4), DI (3)
	Mixed	6	180	54.0	HS (6)
40 s	Male	28	1420	0	CSS (3), HS (1), SMK (24)
	Female	5	876	100	CSS (3), HS (2)
	Mixed	25	1561	51.3	CSS (1), HS (9), HT (2), CKD (4), arthritis (1), after kidney transplantation (2), idiopathic subjective tinnitus (2), spontaneous cervical artery dissection (1), intracranial aneurysm (1), high risk of heart failure (1)
50 s	Male	11	1162	0	CSS (5), HS (2), cardiovascular examination due to stenosis (4)
	Female	6	1230	100	CSS (5), bone mineral density testing/osteoporosis (1)
	Mixed	38	31,418	47.9	CSS (10), HS (12), HT (4), CKD (4), CD (2), SMK (2), hypercholesterolemia (1), systemic sclerosis (1), head and neck cancer (1)
60 s	Male	5	462	0	CSS (4), severe carotid bifurcation occlusive disease (1)
	Female	4	456	100	CSS (4)
	Mixed	34	18,469	44.4	CSS (12), HS (5), HT (3), CKD (6), DI (2), CD (1), left ventricle dysfunction (1), metabolic syndrome (1), cerebrovascular event (1), peripheral arterial disease (1), non-alcoholic fatty liver disease (1)
70 s	Male	1	11	0	CSS (1)
	Female	1	14	100	CSS (1)
	Mixed	7	3086	41.3	CSS (1), HS (2), DI (1), cerebrovascular event (1), aortic valve disease (1), cardiovascular disease (1)

$P_{\textrm{dia}}$, $P_{\textrm{sys}}$, $\textrm{IMT}$, $\textrm{D}$ and *E*, $E_{\textrm{P}}$ seem to increase with age, while $\Delta D$ and $\textrm{DC}$ decrease. However, male and female pooled data for $\textrm{D}$ and *E* do not follow this general trend.

**Fig. 5:**
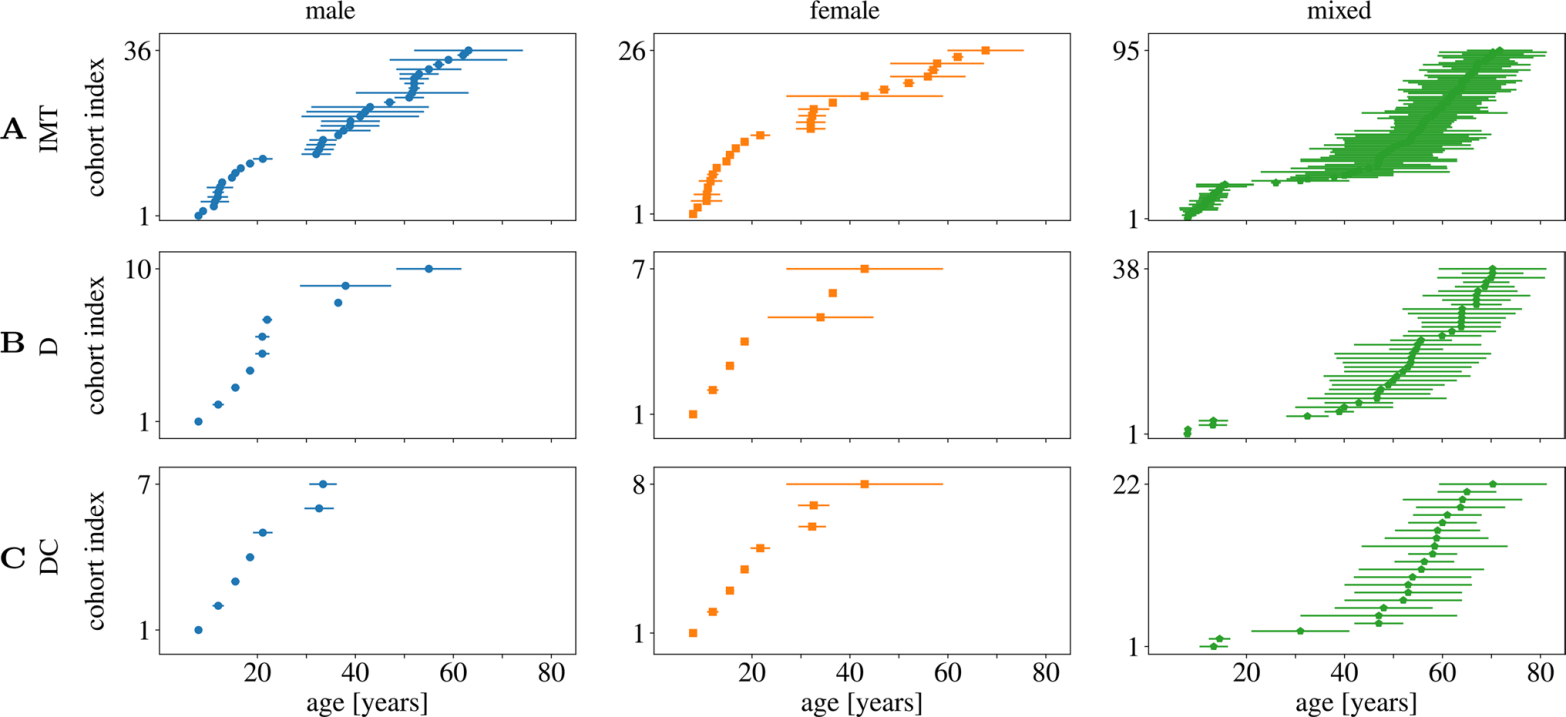
Visualization of age distributions inferred from reported mean (symbols) and standard deviation (line) of participant age for each cohort and measurement of **A** $\text {IMT}$ **B** $\text {D}$ **C** $\text {DC}$ for male, female and mixed data

The mean and a range of $+/-$ one standard deviation of the age per cohort for the parameters $\textrm{IMT}$, $\textrm{D}$, and $\textrm{DC}$ are depicted in Fig. 5. For all parameters, the literature review generally identified a number of studies with narrow age increments in cohorts of children and teenagers, only a few cohorts between the age of 20 and 40, and more cohorts for males than for females. Additionally, apart from the studies including youngest individuals, the age spread within one cohort was relatively large.

### Comparison of literature review data with reference values of a healthy sub-population

**Fig. 6:**
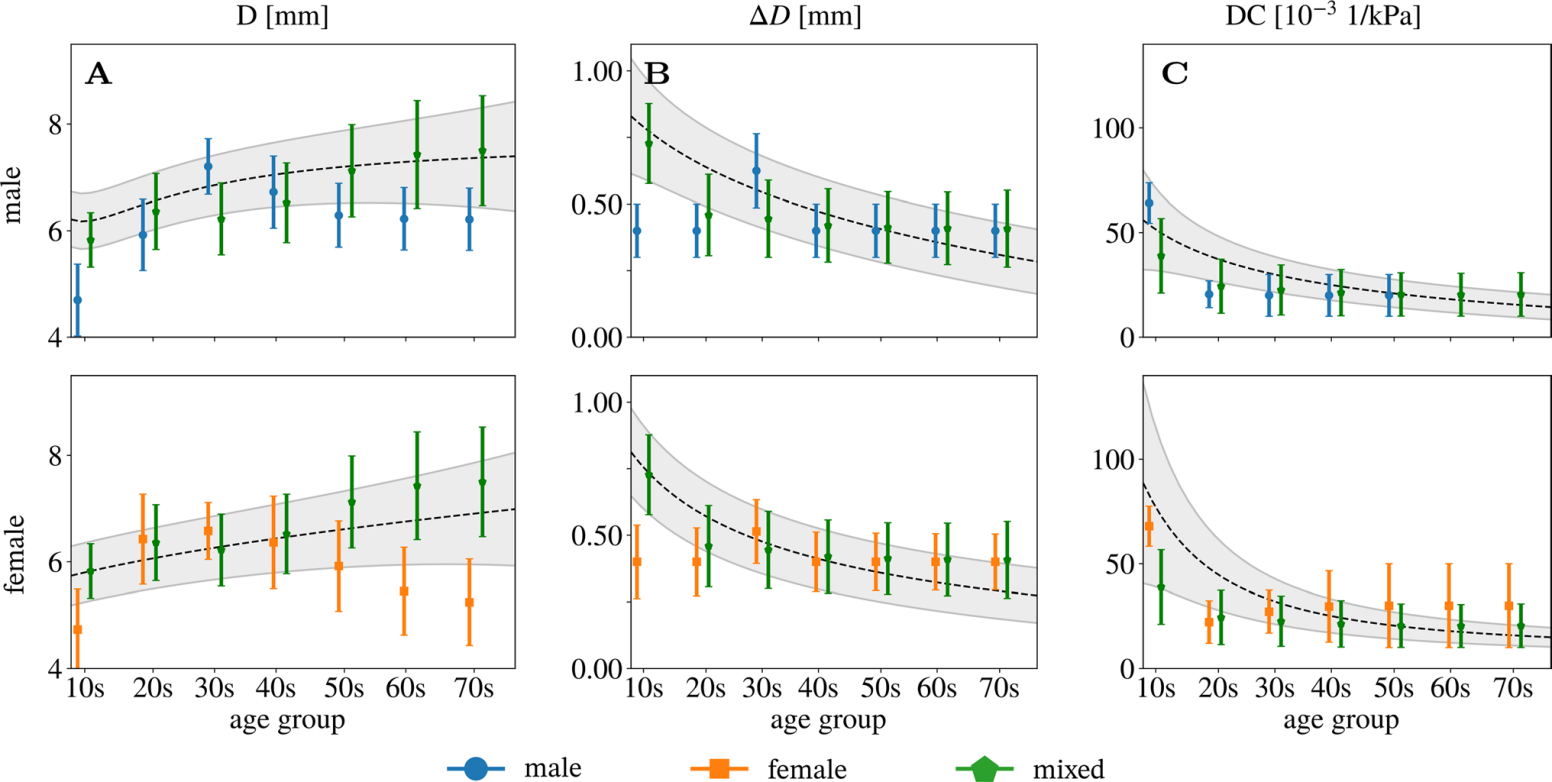
Comparison of **A** lumen diameter *D*, **B** distension $\Delta$D, and **C** distensibility coefficient $\textrm{DC}$ between data from the literature review given as a box plot and a healthy sub-population represented as reference intervals of $\mu \pm \sigma$ retrieved from a parametric regression method based on fractional polynomials (Engelen et al. 1996). The top row shows male (blue circles) and mixed data (green pentagons), and the lower row shows females (orange squares) and mixed data (green pentagons)

Engelen et al. (1996) provided a best fit fractional polynomial for the mean and standard deviation of *D*, $\Delta$*D*, and DC for each sex dependent on age in a healthy sub-population. Their data was based on a total of 3601 individuals from 24 research centres worldwide. Figure 6 shows the comparison of these reference intervals with the pooled and weighted data retrieved from the literature review. There are significant deviations between the reference and the pooled literature data, especially for the younger and the older age groups. Mixed, pooled data follows the trend of the reference intervals more closely, regardless of the sex.

### UQ and SA results

**Fig. 7:**
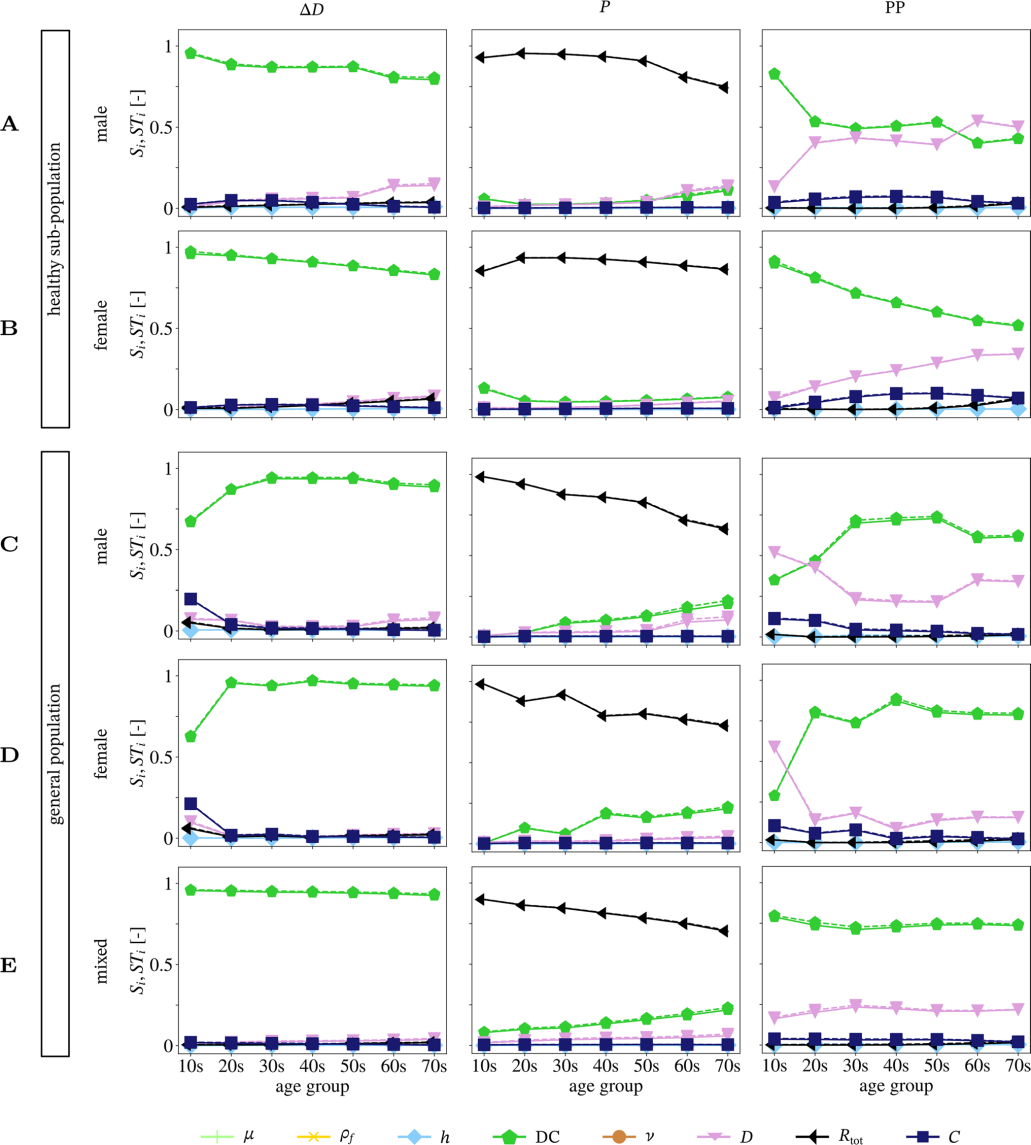
Main ($S_i$ solid line) and total ($\text {ST}_i$ dashed line) sensitivity indices represented over age for each sex group for the QoIs of $\Delta D$ (first column), *P* (second column), and $\textrm{PP}$ (third column). Note that the traces of $\mu$, $\rho$, and $\nu$ have been removed since they are zero. *D* and $\textrm{DC}$ are based on **A** male and **B** female reference values in a healthy sub-population, **C** male and **D** female pooled vales from the literature review, and **E** no sex discrimination based on the literature review

Figure 7 displays the main (solid) and total Sobol (dashed line) indices for the QoIs $\Delta D$, *P*, and PP for each age and sex group. Since $\Delta D$ and *P* are time varying quantities over the cardiac cycle, the sensitivity indices for these QoIs are presented as variance-weighted averages over one cardiac cycle following Eqs. (16) or (17) (Eck et al. 2017). All sensitivity indices are evaluated at the mid point of the vessel. $S_i$ and $\text {ST}_i$ are approximately the same regardless of age, sex, QoI, and whether *D* and $\textrm{DC}$ are based on the literature review of a general population or on reference values of a healthy sub-population. In the following, all trends in the sensitivity indices are described for $S_i$, which implies that $ST_i$ behaves the same as $S_i$. The sensitivity values of $\mu$, $\rho _f$, *h*, and $\nu$ are effectively zero for all QoIs, independent of age, sex, and population groups.

***Comparison of a general versus a healthy sub-population***

Comparing the sensitivity structure of a general versus a healthy sub-population gives for each QoI the following: Diameter change $\Delta D$ is most sensitive to variations in $\textrm{DC}$. In the healthy sub-population, the value of $S_{\textrm{DC}}$ decreases slightly with age while $S_{D}$ increases. In the general population, $\Delta D$ is also most sensitive to variations in $\textrm{DC}$, but in the youngest age groups, there are small sensitivity values for *C*, *D*, and $R_{\textrm{tot}}$ as well.

The pressure *P* in the healthy and general population is most sensitive to variations in $R_{\textrm{tot}}$. There is a small sensitivity value for $\textrm{DC}$ in the youngest age group of the healthy sub-population as well as a slight decay of $S_{R_{\textrm{tot}}}$ with age, which leads to small sensitivity values in *D* and $\textrm{DC}$. In comparison, the general population shows a clear trend where $S_{R_{\textrm{tot}}}$ decreases with age while $S_{\textrm{DC}}$ and $S_{\textrm{D}}$ increase.

The sensitivity of pulse pressure $\textrm{PP}$ shows a clear trend with increasing age for females in a healthy sub-population; $S_{\textrm{DC}}$ decreases, while $S_D$ increases. For the youngest group of the general female population, the most sensitive parameters are in descending order $S_{D}$, $S_{\textrm{DC}}$, and $S_{C}$. All other age groups of the general female population are mainly sensitive to variations in $S_{\text {DC}}$ with small values in $S_D$. The below 20-year-old male, general population group shows similar sensitivity structure as the below 20-year-old group of the general female population with $S_{D}\,<\,S_{\textrm{DC}}\,<\, S_{C}$. All remaining groups of the general male population also have $S_{\text {DC}}\,>\,S_D$, but the difference between the sensitivity values is smaller in the general male population group than in the female counterpart. In contrast to other populations, the youngest group of the healthy male sub-population has a high sensitivity value for $\textrm{DC}$ and a small value for *D*; however, from the 20-year old age group on, $S_{\text {DC}}$ and $S_{D}$ are both around a value of 0.5. Similarly to other populations, in the age groups 20–50 s $S_{\textrm{DC}}\,>\,S_{D}$, but $S_{\textrm{DC}}\,<\,S_{D}$ in the two oldest groups (60 s and 70 s).

***Age-dependent sensitivity structure***

A small age dependence of the sensitivity structure in $\Delta D$ can be seen in the youngest age group of the general population and in the older age groups, where the later effect is mainly present in the healthy sub-population. For parameter variations based on the general mixed-sex population, there is no change in the sensitivity structure of $\Delta D$. There is a clear age dependence for *P* in $S_{R_{\textrm{tot}}}$, where $S_{R_{\text {tot}}}$ decreases, and $S_{\textrm{DC}}$ and $S_{D}$ increase with increasing age. Similarly to $\Delta D$, there is no change in the sensitivity structure of $\text {PP}$ with increasing age for the general mixed-sex population. When sex is discriminated then the sensitivity structure of $\text {PP}$ differs substantially between the youngest age groups and the older age groups. However, the only observed continuous trend of substantial difference was in the healthy female sub-population with $S_{\textrm{DC}}$ decreasing, while $S_D$ increases with age.

**Fig. 8:**
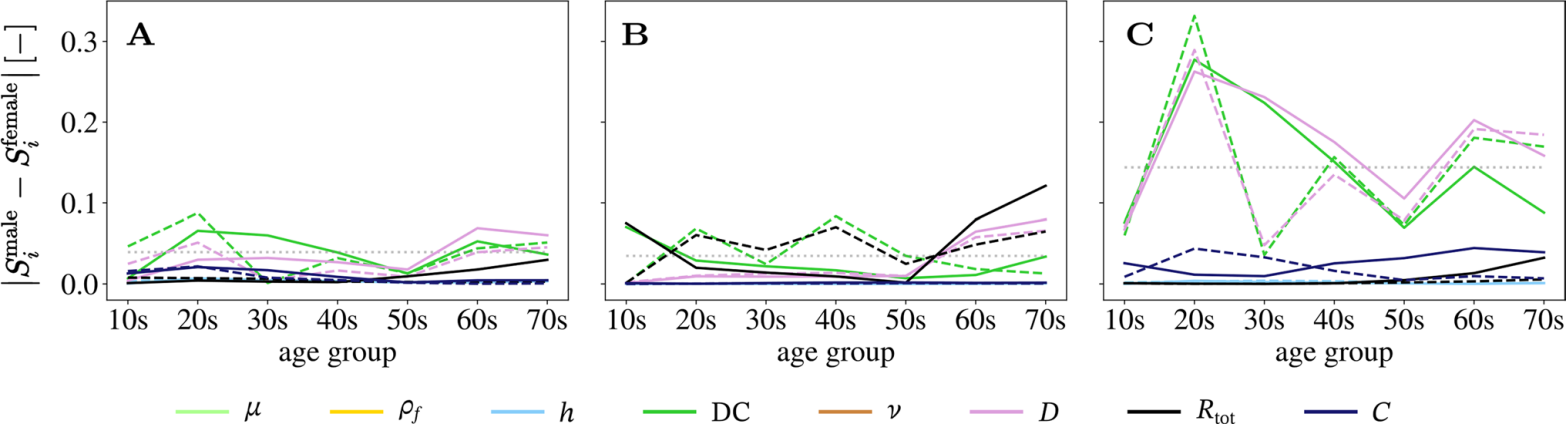
Absolute sex difference in first-order sensitivity indices for the QoIs **a** $\Delta$D **b** *P* and **c** PP when $\textrm{DC}$ and *D* are based on reference values in a healthy sub-population (solid line) and when $\textrm{DC}$ and *D* are based on literature review values (dashed line). Note that the traces of $\mu$, $\rho$, and $\nu$ have been removed since they are zero. In each panel the dotted horizontal line indicates the average sex difference over all age groups in first order sensitivity index of DC of the general population. Numerical values are for $\Delta$D 0.039, *P* 0.035, and PP 0.144

***Sex-dependent sensitivity structure***

$\Delta D$ and *P*’s sensitivity structure shows overall the same trends regardless of sex for all age groups as well as for the healthy and the general sub-population. Absolute sex differences in the total sensitivity indices are shown in Fig. 8. The sensitivity structure of $\textrm{PP}$ indicates sex differences. In the healthy sub-population, $S_{\textrm{DC}}$ decreases and $S_D$ increases continuously for the female group, whereas the value of these indices is approximately the same for the male group. $S_{\textrm{DC}}$ and $S_D$ are also approximately constant for the general population, but the difference between the values of $S_{\textrm{DC}}^{\text {male}}$ and $S_{\textrm{DC}}^{\text {female}}$, as well as $S_{\textrm{D}}^{\text {male}}$ and $S_{\textrm{D}}^{\text {female}}$ is substantial.

**Fig. 9:**
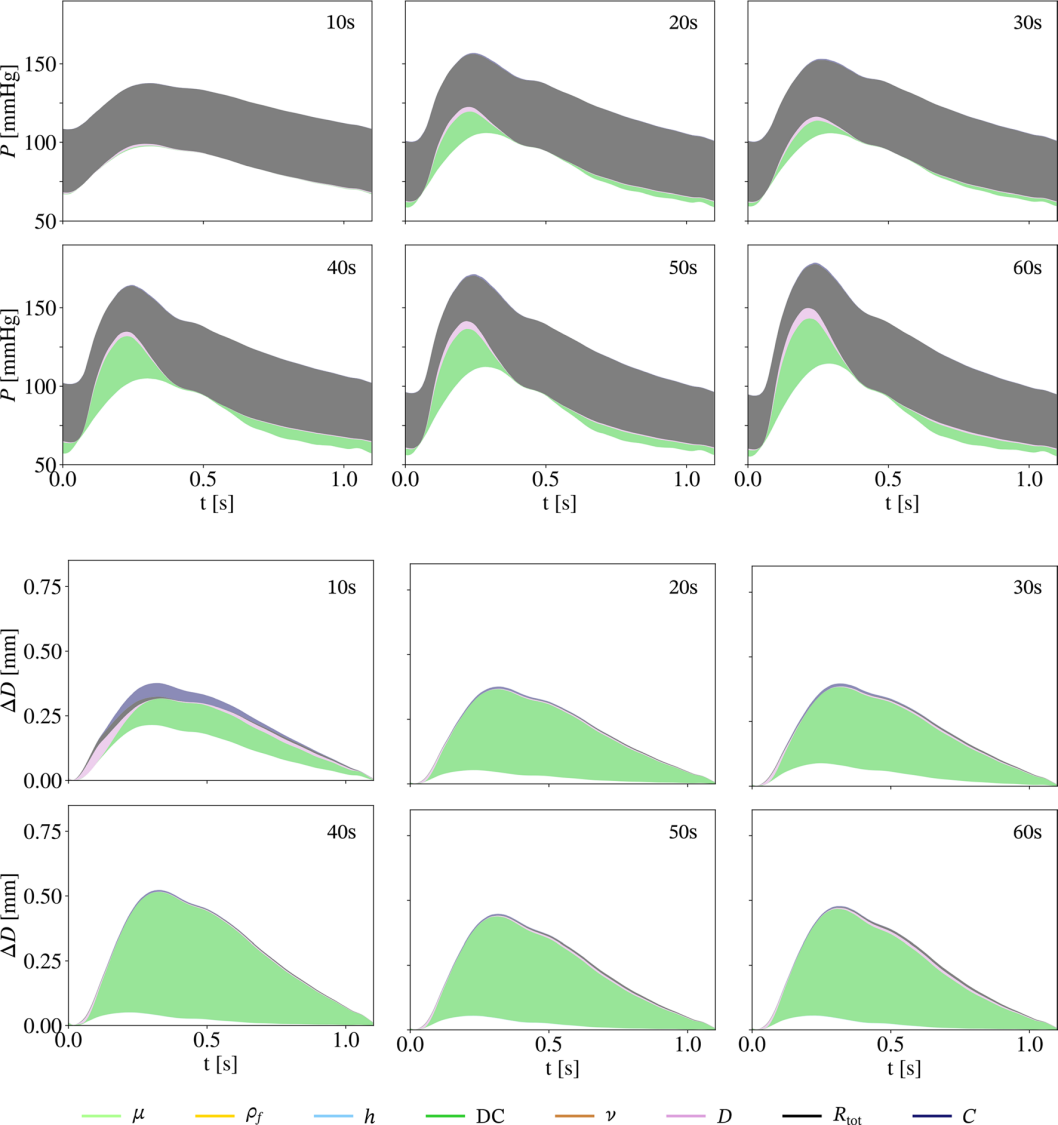
95% prediction interval of pressure *P* in the upper two rows, and diameter change $\Delta D$ in the lower two rows, with partitioned intervals proportional to sensitivity indices. Uncertainties in *D* and $\textrm{DC}$ are based on the pooled female general population. The presented age groups are marked in each panel in the upper right corner

Regardless of basing uncertainties in $\textrm{DC}$ and *D* on reference values of a healthy sub-population or on the pooled values of the literature review, as well as regardless of the sex, the 95% prediction intervals, and the attribution of first-order sensitivity indices are similar within each age group. Therefore, Fig. 9 shows exemplary the 95% prediction interval for *P* and $\Delta D$ of one cardiac cycle for the case where $\textrm{DC}$ and *D* are based on female values in a general population. Panels for $\Delta D$ and *P* in Fig. 7B represent a summary of the case presentation in Fig. 9 through variance-weighted averages of on cardiac cycle. In the 95% prediction interval of *P*, the majority of output variance results from uncertainties in $\textrm{DC}$ and $R_{\textrm{tot}}$ in the Windkessel model. $\textrm{DC}$ contributes only during peak systole and end diastole. With increasing age the contribution of $\textrm{DC}$ to pressure variations increases, especially during systole. The majority of variations in the diameter change are due to uncertainties in $\textrm{DC}$, but in the youngest age group, *C*, $R_{\textrm{tot}}$, and *D* have a small contribution to output variance. The width of the prediction interval decreases with age when variations in $\textrm{DC}$ and *D* are based on reference values of a healthy sub-population, whereas it is the opposite, an increase in width for increasing age, when variations in $\textrm{DC}$ and *D* are based on the literature review. Table 4 shows the average standard deviation of *P* and $\Delta D$ over one cardiac cycle.

**Table 4:** Standard deviation for the QoIs of diameter change $\Delta D$ and pressure *P* averaged over one cardiac cycle, as well as the standard deviation of PP for all simulations

QoI	Data source	Sex	Age group						
			10 s	20 s	30 s	40 s	50 s	60 s	70 s
$\Delta D$ [mm]	Engelen et al. (1996)	Male	0.0327	0.0470	0.0135	0.0133	0.0212	0.0297	0.0176
		Female	0.0253	0.0195	0.0243	0.0271	0.0246	0.0198	0.0215
	Literature review	Male	0.0286	0.0393	0.0211	0.0202	0.0304	0.0308	0.0235
		Female	0.0198	0.0336	0.0328	0.0251	0.0200	0.0363	0.0358
*P* [mmHg]	Engelen et al. (1996)	Male	12.3481	12.8695	11.9883	11.9893	12.2022	12.3210	12.3055
		Female	12.7160	12.2415	12.3272	12.7501	12.4491	12.3421	12.4038
	Literature review	Male	12.8783	13.4013	12.5375	12.5308	13.1350	13.3268	13.2819
		Female	12.6980	14.0248	13.6140	13.7921	12.8582	14.5293	13.9327
PP [mmHg]	Engelen et al. (1996)	Male	8.9140	13.526	4.1970	4.3676	7.1489	8.5225	7.6622
		Female	10.8424	7.4195	8.1091	11.2951	8.9572	8.2579	8.4139
	Literature review	Male	12.1082	15.2814	9.6096	9.0560	13.4678	13.9150	13.5826
		Female	9.9241	17.4042	15.1315	15.6219	10.6950	19.3357	16.4383

## Discussion

In this work, we conducted a structured literature review to determine the distribution of geometric and material parameters for the CCA in different age and sex groups reflecting a general population of both healthy and diseased individuals and without exclusion based on risk factors. Pooled mean values of *D*, $\Delta D$, and $\textrm{DC}$ were compared with reference values based on a healthy sub-population. We pooled mean and standard deviations from each included study weighted by the number of subjects to determine parameter distributions for UQ and SA. Using PC expansion, UQ and SA was performed for each age and sex group on a 1D-CCA model. Additionally, UQ and SA neglecting sex differentiation but including age dependency was conducted.

The inclusion of studies of both general populations, as well as diseased sub-populations, resulted in a sample of population variations more representative of the general population, which is novel compared to previous publications based on healthy sub-populations (Charlton et al. 2019; Engelen et al. 1996). To investigate the influence of uncertainties due to population variations on a numerical model’s prediction, it is important to consider the variations expected in the target population for application of the model. In this context, we envision such a model may be integrated in a general health care setting for screening and data augmentation; thus, variations in a general population are more relevant than the variations only within a healthy sub-population. For the general population, the pooling of the mean geometric and stiffness parameters, Fig. 4, shows increasing trends with age. The ranges retrieved from the literature review are mainly in accordance with a previous summary of literature findings (Charlton et al. 2019). However, there are some differing variations from one age group to the next and between sexes. These results might be due to a generally small number of cohorts covering any specific age and sex group. The literature review identified 239 cohorts from 94 publications for a total of 21 age and sex groups (male, female, mixed).

Comparison of the best fit fractional polynomial for the mean and standard deviation of *D*, $\Delta D$, and $\textrm{DC}$ of a healthy sub-population with the pooled data from the literature review showed some differences (Fig. 6). Note that the best fit fractional polynomial was defined between the ages 15–95, whereas the pooled data includes subjects from the age of 7. Deviations in these parameters for both sexes can be explained by the small number of studies for each age group. The pooled, mixed data follows the best fit reference intervals closely.

A literature review like the one performed in this work can give insights into realistic variations of model parameters for specific age and sex groups or other sub-groups of interest. We recommend basing these variations on a number of publications instead of relying on single study results because we have seen that the reported mean and standard deviations can vary significantly between studies. The data from the literature review can not only be used for UQ and SA, but can also serve as the bases for more advanced statistical analysis to investigate parameter interactions or more precisely characterize the distribution of values, though we note this will in general be very difficult without access to the individual level data or identifying more narrowly focused cohorts.

A limitation of the current literature search has been the restriction to the keyword Young’s modulus as an arterial stiffness measure. Advancing the initial search string with keywords of further stiffness measures significantly increases the amount of results. Including all these studies would increase the robustness of the literature review and probably would even out deviations from the published reference intervals. However, we believe that the overall qualitative result would not be affected by an increased number of studies. In order to include a study in the literature review, the study had to fulfil the eligibility criteria. The inclusion of measurements performed through non-invasive means introduced further uncertainty beyond population variation due to different measurement techniques and operators, but this criteria was necessary to retrieve a large enough sample size to perform UQ and SA on the 1D-model for all groups. It further represents more realistically the variation in data available for a general setting. The analysis was further hampered by incomplete data reporting. Another limitation of the literature review was that the anatomical location of measurement varied over the included studies. Measurements of the left and right CCA were not discriminated and neither was the measurement location from the CCA bifurcation nor the measurement angle considered. The quality of the data could be further improved by data extraction with a second reviewer.

$\text {ST}_i$ quantifies total model output variance due to the variance of parameter $z_i$ and all its interactions with $z_{\sim i}$. In turn, $S_i$ accounts only for $z_i$’s contribution to total output variance. In this work, $S_i$ and $\text {ST}_i$ are approximately the same implying that parameter interactions are not significant. *P* is most sensitive to variations in $R_{\textrm{tot}}$, while $\textrm{PP}$ and $\Delta D$ are most sensitive to variations in $\textrm{DC}$. These results are in line with previous analyses based on local sensitivity analysis (Stergiopulos et al. 1996). A high sensitivity index in $\Delta D$ of $\textrm{DC}$ suggests that it will be possible in an inverse problem to accurately estimate the Young’s modulus from non-invasive CCA distension measurements. Since fluid viscosity $\mu$ and density $\rho _f$, arterial wall thickness *h*, Poisson ratio $\nu$, and Windkessel model compliance *C* have low sensitivity indices, it seems that these parameters do not have a significant influence on model output variability. These results suggest that these parameters ($\mu$, $\rho$, *h*, $\nu$, *C*) can be set to reference values of the respective distributions without changing model output variance while reducing the number of uncertain parameters which need to be explored. However, previous work has shown a relation between the Young’s modulus *E* and the wall thickness *h*. Thus, caution should be taken in setting *h* to a reference value. The same applies to *C* because the uncertainty was assumed to be ± 20% due to a lack of measurements.

The uncertainties based on variation in the general population were typically larger than for those based only on healthy individuals (see Table 4), particularly for pulse pressure. In contrast, the sensitivity structures for $\Delta D$ and *P* were very similar between populations. However, for the youngest age group the ordering of $S_{\textrm{DC}}$, $S_D$, and $S_C$ differs between the general and the healthy sub-population. Further the sensitivities of *P* to variations within the general population showed a slightly stronger age trend. For PP, $S_{\textrm{DC}}$ and $S_D$ were consistently the most sensitive parameters, but the values of sensitivity indices varied between the general and healthy sub-population. Thus, for $\Delta D$ and *P* there are negligible differences between a general and a healthy sub-population, but the population type matters when $\textrm{PP}$ is of interest.

The sensitivity of $\Delta D$ and $\textrm{PP}$ with respect to variations based on mixed-sex cohorts did not exhibit an age dependence, while the sensitivity of *P* showed some dependence on age group. In the female general population, $S_{R_{\text {tot}}}$ decreased from 0.983 to 0.726. Thus, the average standard deviation in pressure due to $R_{\text {tot}}$ reduces from the youngest to the oldest age group by 2.4 mmHg. The sensitivity of $\Delta D$ also showed no age trend in the cases of sex differentiated input parameters, whereas those of $\textrm{PP}$ did. For *P* the sensitivities only had a clear age dependence for the female healthy sub-population. All other age groups have highest sensitivity to $S_{\textrm{DC}}$ and $S_D$ with largely similar values over age, respectively. The exception was in the youngest age groups where the sensitivities differ from this general value. Hence, sensitivity structure does not change with age for $\Delta D$, while it changes for *P* in all populations. The trend with age for $\textrm{PP}$ was dependent on sex and whether the inputs for $\textrm{DC}$ and *D* were based on a general or healthy sub-population.

The uncertainties are generally larger for the female populations than for the male populations, with a few exceptions (see Table 4). The results shown in Fig. 8 suggest only small sex differences for the sensitivity structure in $\Delta D$ and *P* with and average difference in $S_{\text {DC}}$ of 0.039 and 0.035 for $\Delta D$ and *P*, respectively, for the general population. There are also no substantial dissimilarities between the sensitivity structure of $\Delta D$ and *P* for the general and healthy sub-population. When considering $\textrm{PP}$, a difference between the sexes is noticeable and slightly more pronounced for the healthy sub-population than for the general one. The average difference in $S_{\text {DC}}$ is 0.144 for the general population.

*P* at the mid-point of the vessel is very sensitive over the entire cardiac cycle to $R_{\textrm{tot}}$ in the Windkessel model. This result is reasonable since the mean arterial pressure is directly related to the total arterial resistance and the flow as $R_{\text {tot}} = P/Q$. Total arterial compliance regulates $\textrm{PP}$ in the arterial tree. In the presented model, the modelled arterial compliance consists of the compliant vessel and the compliance element in the Windkessel model representing the cerebral vessels. The ratio of vessel to Windkessel compliance is 0.5 in the case of the mixed 40 year old group. $\textrm{DC}$ is a measure of area compliance and determined from $\textrm{PP}$ and *D*, as given in Table 2. Ageing leads to a decrease in $\textrm{DC}$, while *P* in systole becomes more sensitive to variations in $\textrm{DC}$ for increasing age. Further investigations are needed to confirm the arterial compliance distribution between the compliant elements of the CCA and its distal vasculature to clarify their influence on model output variance.

In a physiological and physical sense depends the haemodynamics of the carotid artery highly on the state of the heart, the aorta and total systemic peripheral vasculature; however, the determinants of the pressure for a given inflow are the relationship between local properties of the CCA and the distal vasculature represented by the Windkessel model. Thus, the analysis of the influence of variations in DC is indicative of how much variations of DC locally influence the pressure–flow relationship of the CCA. Nevertheless, the results of these investigations do not show substantial sensitivity of *P* to *C*. Thus, pressure in the CCA cannot be completely explained by the limited scope of this 1D-model, rather, the presented analysis has to be seen in the context of assuming that such a model is applied in a clinical setting where some clinical measurements like carotid inflow and geometry can be determined in a particular physiological state, while assumptions about numerous unmeasured or unmeasurable parameters need to be made. A more comprehensive model may be essential to account for how pathological changes in other regions may drive or compensate what is happening in the CCA. Even though the 1D-model does not fully explain the pressure in the CCA, understanding how model parameters affect model predicted pressure is important when developing procedures to link such a numerical model to clinical data. In particular, the sensitivity of a model’s outputs to particular parameters can be a limiting factor in determining these parameters by adjusting the model to match the data during an inverse problem. Furthermore, the credibility of the model and assumed parameters can be assessed based on whether the variations produced by the model are within a realistic range, i.e. reflective of the expected measurement error or the range of variability for a given physiological state.

The UQ and SA performed in this work is hampered by several factors. Our model considers eight uncertain input parameters which are located in the material and geometric parameters and in the outlet boundary condition. However, even though the flow rate waveform, amplitude, and cycle duration may be directly measured, errors and measurement limitations cause variations which are not accounted for in the present analysis. Spatial changes in diameter, wall thickness, and non-symmetric geometry over the artery have also been neglected. PC expansion assumes that all input parameters in ${\textbf {Z}}$ are independent random variables, but it is likely that *D* and *h* are dependent. Future work could attempt to characterize a statistical dependency structure of the inputs which could then be used for PC with dependent inputs (Mara and Tarantola 2012). Due to lack of knowledge about the variability of cerebral vasculature beds, a variation of ± 20% was assumed for the uncertain parameters in the Windkessel model. This is a small variation around the mean for *C* compared to the relative uncertainty of $\textrm{DC}$ which lay between ± 40–90%. Another assumption within the UQ and SA has been the uniform distribution of the uncertain input parameters. It has been shown that the robustness of the Sobol indices is affected by the distribution of the uncertain input parameters (Hart and Gremaud 2019). Therefore, this assumption’s influence should be subject to further investigations.

## Conclusion

In the present work, we have conducted a structured literature review to characterize the variability of input parameters for a model of the CCA. The analysis aimed to identify distinct distributions associated with specific subgroups delineated by age and sex as the clinical interpretation of physiological parameters can be dependent on an individual’s specific subgroup. These specialized input uncertainties allow UQ and SA to investigate how the model varies for specific subgroups and to ground the interpretation of model predictions within the typical variation of the sub-population. As has been argued, characterization of sensitivity and uncertainty is an essential part of the development and application of computational models of physiology in the clinic (Huberts et al. 2018; Hose et al. 2019). A key part of carrying out UQ and SA is the determination of input uncertainties, and the approach presented in this article is a useful and generalizable way for determining these from prior literature. Of course much more can be done and more advanced statistical models for pooling the data could be employed. However, as an initial means to get representative intervals without cherry-picking, we suggest carrying out such an approach for the relevant population of interest.

In our particular application to a 1D-model of the CCA, we found that *P* is most sensitive to variations in $R_{\textrm{tot}}$ while $\textrm{PP}$ and $\Delta D$ are most sensitive to variations in $\textrm{DC}$, in line with previous analyses based on local sensitivity analysis (Stergiopulos et al. 1996). High sensitivity of $\textrm{DC}$ for $\Delta D$ suggests that accurate estimation of arterial stiffness will be possible during inverse problem inference of the Young’s modulus from non-invasive CCA distension measurements. Variations in $\rho$, $\mu$, and $\nu$ seem to have a negligible effect on QoI ($\Delta D$, *P*, PP) variance under this particular setting such that these parameters can be set to mean values in future investigations of this particular model.

The distributions of diameter and distensibility found for male and female general populations differ somewhat from previously reported reference values for healthy sub-populations and produced higher variability for most sub-groups. The uncertainty of model outputs was higher in the general population in contrast to the results based only on healthy individuals (see Table 4), particularly for pulse pressure. Uncertainty of pulse pressure was typically substantially larger for the female sub-populations than for the male sub-populations, while for diameter change and pressure the differences were minor or of mixed sign. The qualitative sensitivity structure for $\Delta D$, *P*, and PP was largely similar for both populations over age regardless of sex. However, the youngest age group showed differences in sensitivity structure between the two populations which might be due to the age bounds of this group (healthy sub-population 15–19 years; general population 7–19 years). Average sensitivity of the pressure waveform showed a moderate dependence on age, with a decrease of $S_ {R_\text {tot}}$ by 0.257 (accounting for 2.35 mmHg less variation in the oldest group) for the female general population. Sensitivities of PP showed a substantial difference between female and male populations with an average difference between the sexes of 0.144 in $S_{\text {DC}}$ and $S_D$, whereas the average difference in $S_{\text {DC}}$ is 0.039 and 0.035 for $\Delta D$ and *P*, respectively.

As we hypothesized input variability may be population dependent. In the context of modelling the CCA these population dependencies affected our 1D-CCA-model response when considering pressure and pulse pressure, but the sensitivity structure of radial displacement was independent of the considered sub-populations. As the impact will be model and context specific, the approach taken in this paper can serve as a useful method for assessing population specific performance of other computational models.

### Supplementary Information

Below is the link to the electronic supplementary material.

Supplementary file 1 (pdf 132 KB)
