# Supplementary material for: Age and sex-dependent sensitivity analysis of a common carotid artery model
Source: Biomech Model Mechanobiol. 2024 Feb 19;23(3):825–43. doi: 10.1007/s10237-023-01808-0 (PMC11101589; doi:10.1007/s10237-023-01808-0)
Supplement: Supplementary file 1 — (pdf 132 KB) [file 10237_2023_1808_MOESM1_ESM.pdf]

References

[1] Y. Aggoun et al. "Mechanical properties of the common carotid artery in Williams syndrome". In: *Heart* 84.3 (Sept. 2000). Num Pages: 290 Place: London, United Kingdom Publisher: BMJ Publishing Group LTD, p. 290. ISSN: 13556037. DOI: 10.1136/heart.84.3.290. URL: https://www.proquest.com/docview/1780789829/abstract/A8A388291644E7DPQ/1.

[2] Yacine Aggoun et al. "Impaired endothelial and smooth muscle functions and arterial stiffness appear before puberty in obese children and are associated with elevated ambulatory blood pressure". In: *European Heart Journal* 29.6 (Mar. 2008), pp. 792–799. ISSN: 1522-9645, 0195-668X. DOI: 10.1093/eurheartj/ehm633. URL: https://academic.oup.com/eurheartj/article-lookup/doi/10.1093/eurheartj/ehm633.

[3] F. Antonini-Canterin et al. "Arterial stiffness and ventricular stiffness: a couple of diseases or a coupling disease? A review from the cardiologist's point of view". In: *European Journal of Echocardiography* 10.1 (Jan. 1, 2009), pp. 36–43. ISSN: 1525-2167, 1532-2114. DOI: 10.1093/ejechocard/jen236. URL: https://academic.oup.com/ehjcimaging/article-lookup/doi/10.1093/ejechocard/jen236.

[4] Francesco Antonini-Canterin et al. "Echo-Tracking Assessment of Carotid Artery Stiffness in Patients with Aortic Valve Stenosis". In: *Echocardiography* 26.7 (2009), pp. 823–831. ISSN: 1540-8175. DOI: 10.1111/j.1540-8175.2008.00891.x. URL: https://onlinelibrary.wiley.com/doi/abs/10.1111/j.1540-8175.2008.00891.x.

[5] Philippe Arbeille, Romain Provost, and Kathryn Zuj. "Carotid and Femoral Arterial Wall Distensibility During Long-Duration Spaceflight". In: *Aerospace Medicine and Human Performance* 88.10 (Oct. 1, 2017), pp. 924–930. ISSN: 2375-6314. DOI: 10.3357/AMHP.4884.2017. URL: http://www.ingentaconnect.com/content/10.3357/AMHP.4884.2017.

[6] Ricardo L. Armentano et al. "An in vitro study of cryopreserved and fresh human arteries: a comparison with ePTFE prostheses and human arteries studied non-invasively in vivo". In: *Cryobiology* 52.1 (Feb. 1, 2006), pp. 17–26. ISSN: 0011-2240. DOI: 10.1016/j.cryobiol.2005.09.001. URL: https://www.sciencedirect.com/science/article/pii/S0011224005001434.

[7] Donna K Arnett et al. "Variability in ultrasonic measurements of arterial stiffness in the atherosclerosis risk in communities study". In: *Ultrasound in Medicine & Biology* 25.2 (Feb. 1999), pp. 175–180. ISSN: 03015629. DOI: 10.1016/S0301-5629(98)00165-3. URL: https://linkinghub.elsevier.com/retrieve/pii/S0301562998001653.

[8] Anna M. Asp et al. "Aspects of carotid structure and function in health and different stages of chronic kidney disease". In: *Clinical Physiology and Functional Imaging* 38.3 (May 2018), pp. 402–408. ISSN: 14750961. DOI:

10.1111/cpf.12429. URL: https://onlinelibrary.wiley.com/doi/10.1111/cpf.12429.

[9] Michael Barenbrock et al. "Reduced arterial distensibility is a predictor of cardiovascular disease in patients after renal transplantation:" in: *Journal of Hypertension* 20.1 (Jan. 2002), pp. 79–84. ISSN: 0263-6352. DOI: 10.1097/00004872-200201000-00012. URL: http://journals.lww.com/00004872-200201000-00012.

[10] Murat Baykara, Cansu Öztürk, and Filiz Elbüken. "The relationship between bone mineral density and arterial stiffness in women". In: *Diagnostic and Interventional Radiology* 18.5 (Sept. 2012). Num Pages: 441-445 Place: Ankara, Turkey Publisher: Aves Yayincilik Ltd. STI. Section: Cardiovascular Imaging - Original Article, pp. 441–445. ISSN: 13053825. DOI: 10.4261/1305-3825.DIR.5330-11.1. URL: https://www.proquest.com/docview/1676097658/abstract/1F95D2F476C846FFPQ/1.

[11] A. Bjallmark et al. "Ultrasonographic strain imaging is superior to conventional non-invasive measures of vascular stiffness in the detection of age-dependent differences in the mechanical properties of the common carotid artery". In: *European Journal of Echocardiography* 11.7 (Aug. 1, 2010), pp. 630–636. ISSN: 1525-2167, 1532-2114. DOI: 10.1093/ejechocard/jeq033. URL: https://academic.oup.com/ehjcimaging/article-lookup/doi/10.1093/ejechocard/jeq033.

[12] Jacques Blacher et al. "Influence of age and end-stage renal disease on the stiffness of carotid wall material in hypertension". In: *Journal of Hypertension* 17.2 (Feb. 1999), pp. 237–244. ISSN: 0263-6352. URL: https://journals.lww.com/jhypertension/Fulltext/1999/17020/Influence_of_age_and_end_stage_renal_disease_on.8.aspx.

[13] H. Myrthe Boss et al. "Physical Activity and Characteristics of the Carotid Artery Wall in High-Risk Patients—The SMART (Second Manifestations of Arterial Disease) Study". In: *Journal of the American Heart Association* 6.7 (). Publisher: American Heart Association, e005143. DOI: 10.1161/JAHA.116.005143. URL: https://www.ahajournals.org/doi/10.1161/JAHA.116.005143.

[14] Pierre Boutouyrie et al. "Compressibility of the Carotid Artery in Patients With Pseudoxanthoma Elasticum". In: *Hypertension* 38.5 (Nov. 2001). Publisher: American Heart Association, pp. 1181–1184. DOI: 10.1161/hy1101.096108. URL: https://www.ahajournals.org/doi/10.1161/hy1101.096108.

[15] Myrthe van der Bruggen et al. "Pressure-Corrected Carotid Stiffness and Young's Modulus: Evaluation in an Outpatient Clinic Setting". In: *American Journal of Hypertension* 34.7 (Aug. 9, 2021), pp. 737–743. ISSN: 0895-7061, 1941-7225. DOI: 10.1093/ajh/hpab028. URL: https://academic.oup.com/ajh/article/34/7/737/6132012.

[16] Leif Brunvand et al. "Advanced glycation end products in children with type 1 diabetes and early reduced diastolic heart function". In: *BMC Cardiovascular Disorders* 17.1 (Dec. 2017), p. 133. ISSN: 1471-2261. DOI: 10.1186/s12872-017-0551-0. URL: http://bmccardiovascdisord.biomedcentral.com/articles/10.1186/s12872-017-0551-0.

[17] Christopher J. Bulpitt, Chakravarthi Rajkumar, and James D. Cameron. "Vascular Compliance as a Measure of Biological Age". In: *Journal of the American Geriatrics Society* 47.6 (1999), pp. 657–663. ISSN: 1532-5415. DOI: 10.1111/j.1532-5415.1999.tb01586.x. URL: https://onlinelibrary.wiley.com/doi/abs/10.1111/j.1532-5415.1999.tb01586.x.

[18] Caroline Bussy et al. "Intrinsic Stiffness of the Carotid Arterial Wall Material in Essential Hypertensives". In: *Hypertension* 35.5 (May 2000). Publisher: American Heart Association, pp. 1049–1054. DOI: 10.1161/01.HYP.35.5.1049. URL: https://www.ahajournals.org/doi/10.1161/01.HYP.35.5.1049.

[19] Tommy Y. Cai et al. "Carotid extramedial thickness is associated with local arterial stiffness in children". In: *Journal of Hypertension* 34.1 (Jan. 2016), pp. 109–115. ISSN: 0263-6352. DOI: 10.1097/HJH.0000000000000769. URL: https://journals.lww.com/00004872-201601000-00016.

[20] David Calvet et al. "Increased Stiffness of the Carotid Wall Material in Patients With Spontaneous Cervical Artery Dissection". In: *Stroke* 35.9 (Sept. 2004), pp. 2078–2082. ISSN: 0039-2499, 1524-4628. DOI: 10.1161/01.STR.0000136721.95301.8d. URL: https://www.ahajournals.org/doi/10.1161/01.STR.0000136721.95301.8d.

[21] Claudio Carallo et al. "Biphasic hemodynamic effects of LDL-apheresis in common carotid artery". In: (), p. 12.

[22] Cristiana Catena et al. "Decreased fibrinolytic activity is associated with carotid artery stiffening in arterial hypertension". In: *Journal of Research in Medical Sciences* 22.1 (2017), p. 57. ISSN: 1735-1995. DOI: 10.4103/jrms.JRMS_619_16. URL: http://www.jmsjournal.net/text.asp?2017/22/1/57/207292.

[23] M. Chiara Cavallini. "Association of the Auscultatory Gap with Vascular Disease in Hypertensive Patients". In: *Annals of Internal Medicine* 124.10 (May 15, 1996), p. 877. ISSN: 0003-4819. DOI: 10.7326/0003-4819-124-10-199605150-00003. URL: http://annals.org/article.aspx?doi=10.7326/0003-4819-124-10-199605150-00003.

[24] Seraina Caviezel et al. "Variability and reproducibility of carotid structural and functional parameters assessed with transcutaneous ultrasound – Results from the SAPALDIA Cohort Study". In: *Atherosclerosis* 231.2 (Dec. 1, 2013), pp. 448–455. ISSN: 0021-9150. DOI: 10.1016/j.atherosclerosis.2013.10.010. URL: https://www.sciencedirect.com/science/article/pii/S0021915013006102.

[25] W Chen. "Nitric oxide synthase gene polymorphism (G894T) influences arterial stiffness in adults*1The Bogalusa Heart Study". In: *American Journal of Hypertension* 17.7 (July 2004), pp. 553–559. ISSN: 08957061. DOI: 10.1016/j.amjhyper.2004.02.021. URL: https://academic.oup.com/ajh/article-lookup/doi/10.1016/j.amjhyper.2004.02.021.

[26] M. Claridge et al. "Measurement of arterial stiffness in subjects with and without renal disease: Are changes in the vessel wall earlier and more sensitive markers of cardiovascular disease than intima media thickness and pulse pressure?" In: *Indian Journal of Nephrology* 25.1 (2015), p. 21. ISSN: 0971-4065. DOI: 10.4103/0971-4065.138692. URL: http://www.indianjnephrol.org/text.asp?2015/25/1/21/138692.

[27] M. W. Claridge et al. "Measurement of arterial stiffness in subjects with vascular disease: Are vessel wall changes more sensitive than increase in intima–media thickness?" In: *Atherosclerosis* 205.2 (Aug. 1, 2009), pp. 477–480. ISSN: 0021-9150. DOI: 10.1016/j.atherosclerosis.2008.12.030. URL: https://www.sciencedirect.com/science/article/pii/S0021915008009106.

[28] Michele Colaci et al. "The Impaired Elasticity of Large Arteries in Systemic Sclerosis Patients". In: *Journal of Clinical Medicine* 11.12 (Jan. 2022). Number: 12 Publisher: Multidisciplinary Digital Publishing Institute, p. 3256. ISSN: 2077-0383. DOI: 10.3390/jcm11123256. URL: https://www.mdpi.com/2077-0383/11/12/3256.

[29] S. Curcio et al. "High Blood Pressure States in Children, Adolescents, and Young Adults Associate Accelerated Vascular Aging, with a Higher Impact in Females' Arterial Properties". In: *Pediatric Cardiology* 38.4 (Apr. 2017), pp. 840–852. ISSN: 0172-0643, 1432-1971. DOI: 10.1007/s00246-017-1591-z. URL: http://link.springer.com/10.1007/s00246-017-1591-z.

[30] David Della-Morte et al. "Metabolic Syndrome Increases Carotid Artery Stiffness: the Northern Manhattan Study". In: *International Journal of Stroke* 5.3 (June 1, 2010). Publisher: SAGE Publications, pp. 138–144. ISSN: 1747-4930. DOI: 10.1111/j.1747-4949.2010.00421.x. URL: https://doi.org/10.1111/j.1747-4949.2010.00421.x.

[31] Manijhe Mokhtari Dizaji, Mehdi Maerefat, and Saeed Rahgozar. "Estimation of Carotid Artery Pulse Wave Velocity by Doppler Ultrasonography". In: *The Journal of Tehran University Heart Center* 4.2 (2009), pp. 91–96. ISSN: 2008-2371. URL: https://jthc.tums.ac.ir/index.php/jthc/article/view/125.

[32] Abdurrahim Dusak et al. "Arterial distensibility in patients with ruptured and unruptured intracranial aneurysms: Is it a predisposing factor for rupture risk?" In: *Medical Science Monitor* 19 (Aug. 26, 2013). Publisher: International Scientific Information, Inc., pp. 703–709. ISSN: 1234-1010, 1643-3750. DOI: 10.12659/MSM.889032. URL: https://medscimonit.com/abstract/index/idArt/889032.

[33] Anouk L. M. Eikendal et al. "Extracellular vesicle protein CD14 relates to common carotid intima-media thickness in eight year old children". In: *Atherosclerosis* 236.2 (Oct. 1, 2014), pp. 270–276. ISSN: 0021-9150. DOI: 10.1016/j.atherosclerosis.2014.07.018. URL: https://www.sciencedirect.com/science/article/pii/S0021915014012945.

[34] Ulrika Fernberg et al. "Body composition is a strong predictor of local carotid stiffness in Swedish, young adults – the cross sectional Lifestyle, biomarkers, and atherosclerosis study". In: *BMC Cardiovascular Disorders* 19.1 (Dec. 2019), p. 205. ISSN: 1471-2261. DOI: 10.1186/s12872-019-1180-6. URL: https://bmccardiovascdisord.biomedcentral.com/articles/10.1186/s12872-019-1180-6.

[35] G Gamble et al. "Estimation of arterial stiffness, compliance, and distensibility from M-mode ultrasound measurements of the common carotid artery." In: *Stroke* 25.1 (Jan. 1994), pp. 11–16. ISSN: 0039-2499, 1524-4628. DOI: 10.1161/01.STR.25.1.11. URL: https://www.ahajournals.org/doi/10.1161/01.STR.25.1.11.

[36] Bamba Gaye et al. "Ideal Cardiovascular Health and Subclinical Markers of Carotid Structure and Function: The Paris Prospective Study III". In: *Arteriosclerosis, Thrombosis, and Vascular Biology* 36.10 (Oct. 2016), pp. 2115–2124. ISSN: 1079-5642, 1524-4636. DOI: 10.1161/ATVBAHA.116.307920. URL: https://www.ahajournals.org/doi/10.1161/ATVBAHA.116.307920.

[37] Adam D. Gepner et al. "Carotid Artery Longitudinal Displacement, Cardiovascular Disease and Risk Factors: The Multi-Ethnic Study of Atherosclerosis". In: *PLOS ONE* 10.11 (Nov. 6, 2015). Ed. by Xiao-Feng Yang, e0142138. ISSN: 1932-6203. DOI: 10.1371/journal.pone.0142138. URL: https://dx.plos.org/10.1371/journal.pone.0142138.

[38] Adam D. Gepner et al. "Longitudinal Effects of a Decade of Aging on Carotid Artery Stiffness: The Multiethnic Study of Atherosclerosis". In: *Stroke* 45.1 (Jan. 2014), pp. 48–53. ISSN: 0039-2499, 1524-4628. DOI: 10.1161/STROKEAHA.113.002649. URL: https://www.ahajournals.org/doi/10.1161/STROKEAHA.113.002649.

[39] Elisa Cuadrado Godia et al. "Carotid Artery Distensibility". In: *Journal of Ultrasound in Medicine* 26.9 (2007), pp. 1157–1165. ISSN: 1550-9613. DOI: 10.7863/jum.2007.26.9.1157. URL: http://onlinelibrary.wiley.com/doi/abs/10.7863/jum.2007.26.9.1157.

[40] Dorothy M. Gujral et al. "Arterial Stiffness as a Biomarker of Radiation-Induced Carotid Atherosclerosis". In: *Angiology* 67.3 (Mar. 2016), pp. 266–271. ISSN: 0003-3197, 1940-1574. DOI: 10.1177/0003319715589520. URL: http://journals.sagepub.com/doi/10.1177/0003319715589520.

[41] F. Hansen et al. “Diameter and compliance in the human common carotid artery — variations with age and sex”. In: *Ultrasound in Medicine & Biology* 21.1 (Jan. 1, 1995), pp. 1–9. ISSN: 0301-5629. DOI: 10.1016/0301-5629(94)00090-5. URL: https://www.sciencedirect.com/science/article/pii/0301562994000905.

[42] Kevin S. Heffernan et al. “Carotid Artery Stiffness and Hemodynamic Pulsatility During Cognitive Engagement in Healthy Adults: A Pilot Investigation”. In: *American Journal of Hypertension* 28.5 (May 1, 2015), pp. 615–622. ISSN: 0895-7061, 1941-7225. DOI: 10.1093/ajh/hpu198. URL: https://academic.oup.com/ajh/article-lookup/doi/10.1093/ajh/hpu198.

[43] Ronald M.A. Henry et al. “Arterial Stiffness Increases With Deteriorating Glucose Tolerance Status: The Hoorn Study”. In: *Circulation* 107.16 (Apr. 29, 2003), pp. 2089–2095. ISSN: 0009-7322, 1524-4539. DOI: 10.1161/01.CIR.0000065222.34933.FC. URL: https://www.ahajournals.org/doi/10.1161/01.CIR.0000065222.34933.FC.

[44] Concetta Irace et al. “Influence of blood lipids on plasma and blood viscosity”. In: *Clinical Hemorheology & Microcirculation* 57.3 (Sept. 2014). Publisher: IOS Press, pp. 283–290. ISSN: 13860291. DOI: 10.3233/CH-131705. URL: https://search.ebscohost.com/login.aspx?direct=true&db=a9h&AN=97981918&site=ehost-live.

[45] T. Jogestrand, O. Eiken, and J. Nowak. “Relation between the elastic properties and intima-media thickness of the common carotid artery”. In: *Clinical Physiology and Functional Imaging* 23.3 (2003), pp. 134–137. ISSN: 1475-097X. DOI: 10.1046/j.1475-097X.2003.00475.x. URL: https://onlinelibrary.wiley.com/doi/abs/10.1046/j.1475-097X.2003.00475.x.

[46] Jayaraj Joseph et al. “Assessment of Carotid Arterial Stiffness in Community Settings With ARTSENS®”. In: *IEEE Journal of Translational Engineering in Health and Medicine* 9 (2021), pp. 1–11. ISSN: 2168-2372. DOI: 10.1109/JTEHM.2020.3042386. URL: https://ieeexplore.ieee.org/document/9279288/.

[47] Claudia Jourdan et al. “Normative values for intima–media thickness and distensibility of large arteries in healthy adolescents”. In: (2005), p. 9.

[48] S.D.J.M. Kanters et al. “Reproducibility of measurements of intima-media thickness and distensibility in the common carotid artery”. In: *European Journal of Vascular and Endovascular Surgery* 16.1 (July 1998), pp. 28–35. ISSN: 10785884. DOI: 10.1016/S1078-5884(98)80088-9. URL: https://linkinghub.elsevier.com/retrieve/pii/S1078588498800889.

[49] Min Y. Lee et al. “Association Between Hemodynamics in the Common Carotid Artery and Severity of Carotid Atherosclerosis in Patients With Essential Hypertension”. In: *American Journal of Hypertension* 21.7 (July 1, 2008), pp. 765–770. ISSN: 0895-7061. DOI: 10.1038/ajh.2008.182. URL: https://doi.org/10.1038/ajh.2008.182.

[50] Wesley K. Lefferts, Jacqueline A. Augustine, and Kevin S. Heffernan. "Effect of acute resistance exercise on carotid artery stiffness and cerebral blood flow pulsatility". In: *Frontiers in Physiology* 5 (Mar. 19, 2014). ISSN: 1664-042X. DOI: `10.3389/fphys.2014.00101`. URL: `http://journal.frontiersin.org/article/10.3389/fphys.2014.00101/abstract`.

[51] Duanping Liao et al. "Arterial Stiffness and the Development of Hypertension". In: (1999), p. 6.

[52] Jisok Lim et al. "Impact of blood pressure perturbations on arterial stiffness". In: *American Journal of Physiology-Regulatory, Integrative and Comparative Physiology* 309.12 (Dec. 15, 2015). Publisher: American Physiological Society, R1540–R1545. ISSN: 0363-6119. DOI: `10.1152/ajpregu.00368.2015`. URL: `https://journals.physiology.org/doi/full/10.1152/ajpregu.00368.2015`.

[53] Gérard M. London, Bruno Pannier, and Sylvain J. Marchais. "Vascular Calcifications, Arterial Aging and Arterial Remodeling in ESRD". In: *Blood Purification* 35.1 (2013). Publisher: Karger Publishers, pp. 16–21. ISSN: 0253-5068, 1421-9735. DOI: `10.1159/000345172`. URL: `https://www.karger.com/Article/FullText/345172`.

[54] Gérard M. London et al. "Cardiac and arterial interactions in end-stage renal disease". In: *Kidney International* 50.2 (Aug. 1996), pp. 600–608. ISSN: 00852538. DOI: `10.1038/ki.1996.355`. URL: `https://linkinghub.elsevier.com/retrieve/pii/S008525381559649X`.

[55] Afrah E. F. Malik et al. "Single M-Line Is as Reliable as Multiple M-Line Ultrasound for Carotid Artery Screening". In: *Frontiers in Physiology* 12 (Dec. 20, 2021), p. 787083. ISSN: 1664-042X. DOI: `10.3389/fphys.2021.787083`. URL: `https://www.frontiersin.org/articles/10.3389/fphys.2021.787083/full`.

[56] Francesco U.S. Mattace-Raso et al. "Arterial Stiffness and Risk of Coronary Heart Disease and Stroke". In: *Circulation* 113.5 (Feb. 7, 2006). Publisher: American Heart Association, pp. 657–663. DOI: `10.1161/CIRCULATIONAHA.105.555235`. URL: `https://www.ahajournals.org/doi/10.1161/CIRCULATIONAHA.105.555235`.

[57] Roch L. Maurice et al. "Carotid Wall Elastography to Assess Midterm Vascular Dysfunction Secondary to Intrauterine Growth Restriction: Feasibility and Comparison with Standardized Intima-Media Thickness". In: *Ultrasound in Medicine & Biology* 40.5 (May 1, 2014), pp. 864–870. ISSN: 0301-5629. DOI: `10.1016/j.ultrasmedbio.2013.11.013`. URL: `https://www.sciencedirect.com/science/article/pii/S0301562913011782`.

[58] Roch L. Maurice et al. "Noninvasive vascular elastography for carotid artery characterization on subjects without previous history of atherosclerosis". In: *Medical Physics* 35.8 (2008), pp. 3436–3443. ISSN: 2473-4209. DOI: `10.1118/1.2948320`. URL: `https://onlinelibrary.wiley.com/doi/abs/10.1118/1.2948320`.

[59] Beatrix Mersich et al. "Transposition of Great Arteries Is Associated With Increased Carotid Artery Stiffness". In: *Hypertension* 47.6 (June 2006), pp. 1197–1202. ISSN: 0194-911X, 1524-4563. DOI: 10.1161/01.HYP.0000218826.72592.e9. URL: https://www.ahajournals.org/doi/10.1161/01.HYP.0000218826.72592.e9.

[60] Jean-Jacques Mourad et al. "Increased Stiffness of Radial Artery Wall Material in End-Stage Renal Disease". In: *Hypertension* 30.6 (Dec. 1997). Publisher: American Heart Association, pp. 1425–1430. DOI: 10.1161/01.HYP.30.6.1425. URL: https://www.ahajournals.org/doi/10.1161/01.HYP.30.6.1425.

[61] Yoji Nagai et al. "Carotid arterial stiffness as a surrogate for aortic stiffness: relationship between carotid artery pressure–strain elastic modulus and aortic pulse wave velocity". In: *Ultrasound in Medicine & Biology* 25.2 (Feb. 1999), pp. 181–188. ISSN: 03015629. DOI: 10.1016/S0301-5629(98)00146-X. URL: https://linkinghub.elsevier.com/retrieve/pii/S030156299800146X.

[62] Nicola Napoli et al. "Increased Carotid Thickness in Subjects with Recently-Diagnosed Diabetes from Rural Cameroon". In: *PLoS ONE* 7.8 (Aug. 20, 2012). Ed. by Giorgio Sesti, e41316. ISSN: 1932-6203. DOI: 10.1371/journal.pone.0041316. URL: https://dx.plos.org/10.1371/journal.pone.0041316.

[63] Francisco Núñez et al. "Carotid Artery Stiffness as an Early Marker of Vascular Lesions in Children and Adolescents With Cardiovascular Risk Factors". In: *Revista Española de Cardiología (English Edition)* 63.11 (Jan. 2010), pp. 1253–1260. ISSN: 18855857. DOI: 10.1016/S1885-5857(10)70250-4. URL: https://linkinghub.elsevier.com/retrieve/pii/S1885585710702504.

[64] Ebenezer Oni et al. "Nonalcoholic Fatty Liver Disease Is Associated With Arterial Distensibility and Carotid Intima-Media Thickness: (from the Multi-Ethnic Study of Atherosclerosis)". In: *The American Journal of Cardiology* 124.4 (Aug. 2019), pp. 534–538. ISSN: 00029149. DOI: 10.1016/j.amjcard.2019.05.028. URL: https://linkinghub.elsevier.com/retrieve/pii/S0002914919305958.

[65] Deepa Pandit et al. "Carotid arterial stiffness in overweight and obese Indian children". In: *Journal of Pediatric Endocrinology and Metabolism* 24.1 (Jan. 1, 2011). ISSN: 2191-0251, 0334-018X. DOI: 10.1515/jpem.2011.086. URL: https://www.degruyter.com/document/doi/10.1515/jpem.2011.086/html.

[66] Michał Podgórski et al. "Peripheral vascular stiffness, assessed with two-dimensional speckle tracking versus the degree of coronary artery calcification, evaluated by tomographic coronary artery calcification index". In: *Archives of Medical Science* 1 (2015), pp. 122–129. ISSN: 1734-1922. DOI: 10.5114/aoms.2015.49205. URL: http://www.termedia.pl/doi/10.5114/aoms.2015.49205.

[67] Christopher J. A. Pugh et al. “Carotid artery wall mechanics in young males with high cardiorespiratory fitness”. In: *Experimental Physiology* 103.9 (Sept. 2018), pp. 1277–1286. ISSN: 09580670. DOI: 10.1113/EP087067. URL: http://doi.wiley.com/10.1113/EP087067.

[68] Mehravar Rafati et al. “Analysis of sequential ultrasound frames for the measurement of hemodynamic stresses, critical bent buckling pressure, and critical buckling torque of human common carotid atherosclerosis”. In: *Clinical Biomechanics* 87 (July 2021), p. 105401. ISSN: 02680033. DOI: 10.1016/j.clinbiomech.2021.105401. URL: https://linkinghub.elsevier.com/retrieve/pii/S0268003321001315.

[69] Jean Rossario Raj, Smk Rahman, and Sneh Anand. “An insight into elasticity analysis of common carotid artery using ultrasonography”. In: *Proceedings of the Institution of Mechanical Engineers, Part H: Journal of Engineering in Medicine* 230.8 (Aug. 2016), pp. 750–760. ISSN: 0954-4119, 2041-3033. DOI: 10.1177/0954411916650220. URL: http://journals.sagepub.com/doi/10.1177/0954411916650220.

[70] F. Rakebrandt et al. “Arterial Wave Intensity and Ventricular-Arterial Coupling by Vascular Ultrasound: Rationale and Methods for the Automated Analysis of Forwards and Backwards Running Waves”. In: *Ultrasound in Medicine & Biology* 35.2 (Feb. 1, 2009), pp. 266–277. ISSN: 0301-5629. DOI: 10.1016/j.ultrasmedbio.2008.08.013. URL: https://www.sciencedirect.com/science/article/pii/S0301562908003840.

[71] Reino O. Raninen, Markku M. Kupari, and Pauli E. Hekali. “Carotid and femoral artery stiffnessin Takayasu’s arteritis”. In: *Scandinavian Journal of Rheumatology* 31.2 (Jan. 2002), pp. 85–88. ISSN: 0300-9742, 1502-7732. DOI: 10.1080/030097402529375 95. URL: http://www.tandfonline.com/doi/full/10.1080/03009740252937595.

[72] W A Riley et al. “Ultrasonic measurement of the elastic modulus of the common carotid artery. The Atherosclerosis Risk in Communities (ARIC) Study.” In: *Stroke* 23.7 (July 1992), pp. 952–956. ISSN: 0039-2499, 1524-4628. DOI: 10.1161/01.STR.23.7.952. URL: https://www.ahajournals.org/doi/10.1161/01.STR.23.7.952.

[73] Alexander J. Rosenberg et al. “Healthy aging and carotid performance: strain measures and β-stiffness index”. In: *Hypertension Research* 41.9 (Sept. 2018), pp. 748–755. ISSN: 0916-9636, 1348-4214. DOI: 10.1038/s41440-018-0065-x. URL: http://www.nature.com/articles/s41440-018-0065-x.

[74] Å. Rydén Ahlgren et al. “Female gender increases stiffness of elastic but not of muscular arteries in type I diabetic patients”. In: *Clinical Physiology and Functional Imaging* 22.6 (2002), pp. 409–415. ISSN: 1475-097X. DOI: 10.1046/j.1475-097X.2002.00451.x. URL: https://onlinelibrary.wiley.com/doi/abs/10.1046/j.1475-097X.2002.00451.x.

[75] Åsa Rydén Ahlgren et al. "Dynamic behaviour of the common femoral artery: age and gender of minor importance". In: *Ultrasound in Medicine & Biology* 27.2 (Feb. 1, 2001), pp. 181–188. ISSN: 0301-5629. DOI: 10.1016/S0301-5629(00)00339-2. URL: https://www.sciencedirect.com/science/article/pii/S0301562900003392.

[76] Paolo Salvi et al. "Non-Invasive Assessment of Arterial Stiffness: Pulse Wave Velocity, Pulse Wave Analysis and Carotid Cross-Sectional Distensibility: Comparison between Methods". In: *Journal of Clinical Medicine* 11.8 (Apr. 15, 2022), p. 2225. ISSN: 2077-0383. DOI: 10.3390/jcm11082225. URL: https://www.mdpi.com/2077-0383/11/8/2225.

[77] M Satija et al. "Endothelial function, arterial wall mechanics and intima media thickness in juvenile idiopathic arthritis". In: (2014), p. 8.

[78] Kelly M. T. Schmidt et al. "Longitudinal Effects of Cigarette Smoking and Smoking Cessation on Aortic Wave Reflections, Pulse Wave Velocity, and Carotid Artery Distensibility". In: *Journal of the American Heart Association* 8.24 (Dec. 17, 2019), e013939. ISSN: 2047-9980. DOI: 10.1161/JAHA.119.013939. URL: https://www.ahajournals.org/doi/10.1161/JAHA.119.013939.

[79] Robert H Selzer et al. "Improved common carotid elasticity and intima-media thickness measurements from computer analysis of sequential ultrasound frames". In: *Atherosclerosis* 154.1 (Jan. 1, 2001), pp. 185–193. ISSN: 0021-9150. DOI: 10.1016/S0021-9150(00)00461-5. URL: https://www.sciencedirect.com/science/article/pii/S0021915000004615.

[80] Neslihan Seyrek et al. "Which Parameter Is More Influential on the Development of Arteriosclerosis in Hemodialysis Patients?" In: *Renal Failure* 25.6 (Jan. 2003), pp. 1011–1018. ISSN: 0886-022X, 1525-6049. DOI: 10.1081/JDI-120026036. URL: http://www.tandfonline.com/doi/full/10.1081/JDI-120026036.

[81] Manish D. Sinha et al. "Decreased Arterial Elasticity in Children With Nondialysis Chronic Kidney Disease Is Related to Blood Pressure and Not to Glomerular Filtration Rate". In: *Hypertension* 66.4 (Oct. 2015), pp. 809–815. ISSN: 0194-911X, 1524-4563. DOI: 10.1161/HYPERTENSIONAHA.115.05516. URL: https://www.ahajournals.org/doi/10.1161/HYPERTENSIONAHA.115.05516.

[82] Midori Tanaka et al. "Intermittent, moderate-intensity aerobic exercise for only eight weeks reduces arterial stiffness: evaluation by measurement of stiffness parameter and pressure–strain elastic modulus by use of ultrasonic echo tracking". In: *Journal of Medical Ultrasonics* 40.2 (Apr. 1, 2013), pp. 119–124. ISSN: 1613-2254. DOI: 10.1007/s10396-012-0408-1. URL: https://doi.org/10.1007/s10396-012-0408-1.

[83] Yacob G. Tedla et al. "Association between long-term blood pressure control and ten-year progression in carotid arterial stiffness among hypertensive individuals: the multiethnic study of atherosclerosis". In: *Journal of Hypertension* 35.4 (Apr. 2017), pp. 862–869. ISSN: 0263-6352. DOI: 10.1097/HJH.0000000000001199. URL: https://journals.lww.com/00004872-201704000-00028.

[84] Shih-Te Tu et al. "Carotid Intima-Media Thickness and Stiffness Are Independent Risk Factors for Atherosclerotic Diseases". In: *Journal of Investigative Medicine* 58.6 (Aug. 2010). Num Pages: 786 Place: London, United Kingdom Publisher: BMJ Publishing Group LTD, p. 786. ISSN: 10815589. DOI: https://doi.org/10.2310/JIM.0b013e3181e8019d. URL: https://www.proquest.com/docview/1786924048/abstract/F7BB4A49EAC8404DPQ/1.

[85] Elaine M. Urbina et al. "Youth With Obesity and Obesity-Related Type 2 Diabetes Mellitus Demonstrate Abnormalities in Carotid Structure and Function". In: *Circulation* 119.22 (June 9, 2009), pp. 2913–2919. ISSN: 0009-7322, 1524-4539. DOI: 10.1161/CIRCULATIONAHA.108.830380. URL: https://www.ahajournals.org/doi/10.1161/CIRCULATIONAHA.108.830380.

[86] Olga Vriz et al. "Comparison of arterial stiffness/compliance in the ascending aorta and common carotid artery in healthy subjects and its impact on left ventricular structure and function". In: *The International Journal of Cardiovascular Imaging* 33.4 (Apr. 2017), pp. 521–531. ISSN: 1569-5794, 1573-0743. DOI: 10.1007/s10554-016-1032-8. URL: http://link.springer.com/10.1007/s10554-016-1032-8.

[87] Yonghuai Wang et al. "Incremental value of carotid elasticity modulus using shear wave elastography for identifying coronary artery disease in patients without carotid plaque". In: *Journal of Hypertension* 39.6 (June 2021), pp. 1210–1220. ISSN: 0263-6352, 1473-5598. DOI: 10.1097/HJH.0000000000002773. URL: https://journals.lww.com/10.1097/HJH.0000000000002773.

[88] Heidi Weberruß et al. "Increased intima-media thickness is not associated with stiffer arteries in children". In: *Atherosclerosis* 242.1 (Sept. 2015), pp. 48–55. ISSN: 00219150. DOI: 10.1016/j.atherosclerosis.2015.06.045. URL: https://linkinghub.elsevier.com/retrieve/pii/S0021915015300071.

[89] Heidi Weberruß et al. "Reduced arterial stiffness in very fit boys and girls". In: *Cardiology in the Young* 27.1 (Jan. 2017), pp. 117–124. ISSN: 1047-9511, 1467-1107. DOI: 10.1017/S1047951116000226. URL: https://www.cambridge.org/core/product/identifier/S1047951116000226/type/journal_article.

[90] Luca Zanoli et al. "Increased carotid stiffness and remodelling at early stages of chronic kidney disease". In: *Journal of Hypertension* 37.6 (June 2019), pp. 1176–1182. ISSN: 0263-6352. DOI: 10.1097/HJH.0000000000002007. URL: https://journals.lww.com/00004872-201906000-00010.

[91] Pu Zhang et al. "Echo-tracking evaluation of changes in common carotid artery wall elasticity after smoking cessation". In: *Journal of Clinical Ultrasound* 47.3 (Mar. 2019), pp. 144–149. ISSN: 00912751. DOI: 10.1002/jcu.22669. URL: https://onlinelibrary.wiley.com/doi/10.1002/jcu.22669.

[92] Pu Zhang et al. "Effect of Smoking on Common Carotid Artery Wall Elasticity Evaluated by Echo Tracking Technique". In: *Ultrasound in Medicine & Biology* 40.3 (Mar. 1, 2014), pp. 643–649. ISSN: 0301-5629. DOI: 10.1016/j.ultrasmedbio.2013.10.009. URL: https://www.sciencedirect.com/science/article/pii/S0301562913010776.

[93] Concetta Zito et al. "Interplay between arterial stiffness and diastolic function: a marker of ventricular–vascular coupling". In: *Journal of Cardiovascular Medicine* 15.11 (Nov. 2014), pp. 788–796. ISSN: 1558-2027. DOI: 10.2459/JCM.0000000000000093. URL: http://journals.lww.com/01244665-201411000-00002.

[94] Zhiyong Zou et al. "High serum level of lutein may be protective against early atherosclerosis: The Beijing atherosclerosis study". In: *Atherosclerosis* 219.2 (Dec. 1, 2011), pp. 789–793. ISSN: 0021-9150. DOI: 10.1016/j.atherosclerosis.2011.08.006. URL: https://www.sciencedirect.com/science/article/pii/S0021915011007556.
